# Cytoprotective role of octacosanol in lipopolysaccharide-induced inflammation

**DOI:** 10.3389/fimmu.2026.1770191

**Published:** 2026-02-11

**Authors:** Jingrong Tang, Zhi-Hong Yang, Wenling Li, Huaitian Liu, Diego Lucero, Denis Sviridov, Olivia White, Julia Kun, Yoh-suke Mukouyama, Alan T. Remaley

**Affiliations:** 1 Lipoprotein Metabolism Laboratory, National Heart, Lung and Blood Institute (NHLBI), National Institutes of Health (NIH), Bethesda, MD, United States; 2 Laboratory of Stem Cell and Neuro-Vascular Biology, Cell and Development Biology Center, National Heart, Lung and Blood Institute (NHLBI), National Institutes of Health (NIH), Bethesda, MD, United States; 3 Laboratory of Human Carcinogenesis, National Cancer Institute, National Institutes of Health (NIH), Bethesda, MD, United States

**Keywords:** human aortic endothelial cells, cell-cell junctions, endothelial integrity, inflammation, monocyte adhesion, octacosanol, policosanol

## Abstract

Inflammation and endothelial dysfunction are key steps in the pathogenesis of atherosclerosis. Octacosanol (C_28_H_58_O, OCT), a very-long-chain saturated aliphatic alcohol (VLCA) with 28 carbons, is the main component of policosanol, a nutraceutical mixture of VLCAs (C20-C34) extracted from plants. Polycosanol in animal models is known to reduce atherosclerosis, but its mechanism of action remains unclear. This study investigates the pathways by which OCT alleviates LPS-induced inflammation in primary human aortic endothelial cells (HAECs). After overnight pretreatment with purified OCT, inflammation in HAECs was induced by lipopolysaccharide (LPS) at 100 ng/ml. The effects of OCT on the levels of pro-inflammatory cytokines, and molecules involved in inflammation signaling pathway, cell adhesion, and cell integrity were examined using quantitative RT-PCR, enzyme-linked immunosorbent assay, flow cytometry, or confocal microscopy in LPS-stimulated HAECs. The group of untreated HAECs was used as a control. OCT pretreatment of HAECs significantly reduced LPS-induced inflammatory responses, decreasing levels of IL-6, IL-8, and MCP-1 mRNA and protein, as well as TLR4, MYD88, TIRAP, TRAF6, and IRAK1 mRNA (p < 0.05). In a monocyte adhesion assay, LPS exposure increased human monocytic cells (THP-1) adherence to HAECs, whereas OCT pretreatment suppressed the LPS-induced adhesion of THP-1 to HAECs in a time- and dose-dependent manner, by blocking the mRNA and protein expression of adhesion molecules (VCAM-1, ICAM-1, P- and E-SELECTIN) (p < 0.05). OCT also suppressed mRNA and protein levels of CORTACTIN, VINCULIN, and TALIN, and inhibited focal adhesion and lamellipodia formation, cell deformation, and migration in response to LPS (p < 0.05). In addition, an endothelial permeability assay revealed that OCT pretreatment also improved endothelial cell integrity after LPS stimulation by preserving both adherens junctions and tight junctions formed by VE-CADHERIN, β -CATENIN, and Zonula Occludens-1 (ZO-1) (p < 0.05). In summary, the multiple cytoprotective effects of OCT against LPS-induced inflammation in endothelial cells could contribute to the anti-atherogenic properties of policosanol.

## 1 Introduction

Atherosclerotic cardiovascular disease (ASCVD) is a leading cause of death and disability throughout the world (1). Inflammation plays a fundamental role in all stages of atherosclerosis development, from its initiation to the final rupture of advanced plaques (2). Inflammatory activation of the endothelium triggers monocytes binding to endothelial cells and their migration into the vessel wall through a multi-step process, involving different adhesion molecules, such as selectins, intercellular adhesion molecule-1 (ICAM-1) and Vascular cell adhesion molecule-1 (VCAM-1) (3, 4). Inflammation also triggers increased vascular permeability (5). Inter-endothelial cell junctions, including adherens junctions and tight junctions, regulate cell-to-cell adhesion and play a crucial role in vascular permeability (6). Inflammation acts on endothelium to reorganize the cytoskeleton, decreasing the synthesis and expression of inter-endothelial junction proteins (7). This can lead to the increased infiltration of LDL, another major driver of atherosclerosis, into the vessel wall and the formation of foam cells by macrophage-derived monocytes (5, 8).

There is growing interest in the use of various nutraceuticals for the prevention of a wide variety of diseases (9, 10). Nutraceuticals containing fatty acids or other lipid-like molecules have been shown to improve inflammation and vascular endothelial cell function (11–14). Policosanol, an over-the-counter nutraceutical supplement, is a mixture of long-chain saturated aliphatic alcohols (C20-34) purified from natural sources like sugarcane wax, rice bran, beeswax, and fruits (15). In addition to its possible cholesterol-lowering effect (16), polycosanol was also shown to stabilize plaques, reduce macrophage infiltration and improve endothelial cell function (17–20). Octacosanol (OCT; C_28_H_58_O), an aliphatic 28-carbon primary aliphatic alcohol, is the main lipid component in polycosanol (approximately 60% by weight) (21). Several animal studies have shown that OCT can improve the lipid profile and favorably alter cellular redox status (22–24). In a mouse model of colitis, OCT showed potent anti-inflammatory effects in macrophages by inhibiting proinflammatory signaling pathways, involving MAPK and NF-κB (25).

We investigated in the current study the cytoprotective effect of OCT on primary human aortic endothelial cells (HAECs) from inflammation. Multiple anti-inflammatory effects were observed for OCT after lipopolysaccharide (LPS) treatment of HAECs, thereby improving our understanding of the possible health benefits of policosanol supplementation.

## 2 Materials and methods

### 2.1 Reagents

OCT (> 99% GC purity; Sigma-Aldrich, Saint Louis, MO, USA) was dissolved in hot ethanol, diluted with fetal bovine Serum (FBS)-free Dulbecco’s Modified Eagle Medium (DMEM) (Thermo Fisher Scientific, MA, USA), aliquoted and stored at -20°C. Purified Lipopolysaccharide (LPS, γ-irradiated, BioXtra, suitable for cell culture; Sigma-Aldrich) extracted from *Escherichia coli* 026:B6 was dissolved in sterile distilled water, aliquoted and stored at -20°C. Cells were counted with the Counting Kit-8 (Dojindo Molecular Technologies, Rockville, MD, USA). Cytotrace™ Green CMFDA dye, fluorescein isothiocyanate (FITC)-dextran and Image-iT™ Fixative Solutions and goat serum were purchased from Thermo Fisher Scientific. Fluor™ 568 carboxylic acid, succinyl ester was purchased from Molecular Probes (OR, USA). Total RNA was extracted using a Qiagen RNeasy^®^ Mini Kit (Qiagen, Hilden, Germany). Both iScript cDNA Synthesis Kit and iTaq Universal Probes Supermix were from Bio-Rad Laboratories (CA, USA). All antibodies used in this study are listed in **Table 1**.

**Table 1:** List of primary antibodies used in confocal imaging analysis.

Primary antibodies	Company	Catalog number	Dilution
VCAM-1	Thermo Fisher Scientific	MA5-16429	1:400
ICAM-1	Thermo Fisher Scientific	14-0549-82	1:400
E-selectin	Thermo Fisher Scientific	MA1-06507	1:400
P-selectin	Thermo Fisher Scientific	701257	1:400
ZO-1	Thermo Fisher Scientific	33-9100	1:800
ZO-2	Thermo Fisher Scientific	37-4700	1:500
Talin	Huabio	HA723164	1:500
Cortactin	Huabio	ET1610-87	1:200
Vinculin	Genetex	GTX637857	1:500
VE-Cadherin	Abcam	Ab33168	1:1000
β-Catenin	Protein Tech	66379-1-Ig	1:500

### 2.2 Cell culture

Primary human aortic endothelial cells (HAECs) were purchased from ScienCell Research Laboratories (San Diego, CA, USA) and grown in Endothelial Cell Medium (ECM) (ScienCell Research Lab) supplemented with 5% FBS (Thermo Fisher Scientific), 1% of Endothelial Cell Growth Supplement and 1% of penicillin/streptomycin solution at 37°C in a humidified atmosphere of 95% air and 5% CO_2_. The ECM was changed every other day until the cells reached confluence. Only HAECs at passages between 4–5 were used for all experiments (26, 27).

### 2.3 Cell viability assay

HAECs in the logarithmic growth phase were disassociated with trypsinization, centrifuged, and seeded into 96-well flat-bottom plates at a density of 5 × 10^4^ cells/mL. The cells were cultured in endothelial growth medium and incubated overnight at 37°C in a humidified atmosphere of 5% CO_2_. To achieve cell cycle synchronization at the G0/G1 phase, the medium was replaced with serum-free DMEM and the cells were incubated for an additional overnight period. Following synchronization, cells were treated with various concentrations of OCT prepared in DMEM supplemented with 2% lipoprotein-depleted FBS (LD-FBS) and maintained at 37°C with 5% CO_2_ for 48 h. LD-FBS was prepared from commercial FBS, using a traditional lipoprotein separation procedure (28). Briefly, FBS was adjusted to a density of 1.21 g/mL with potassium Bromide (KBr, Sigma-Aldrich) and subjected to ultracentrifugation at 330,000 × g for 48 h at 4°C. The upper lipoprotein-enriched fraction was discarded, and the bottom fraction was extensively dialyzed against PBS at 4°C to remove residual KBr and stored at -20 °C. After treatment, the medium in each well was replaced with 100 μL of fresh medium containing 10 μL of CCK-8 reagent (Dojindo) and incubated for 4 h at 37°C. Absorbance was measured at 420 nm using a microplate reader (Bio-Tek Synergy H1, Vermont, USA). Cell viability in the treated groups was expressed relative to the untreated control group. Data represent the mean from four independent experiments, with each experiment performed at least three times.

### 2.4 THP-1 cell adhesion assay

HAECs were seeded into 12-well plates at a density of 5.0 × 10^4^ cells/mL and incubated overnight at 37°C in a humidified atmosphere containing 5% CO_2_. The following day, HAECs were treated with varying concentrations of OCT (0, 1.25, 2.5, or 5μM) and incubated overnight under the same conditions. To induce inflammation, HAECs were stimulated with 100 ng/mL of LPS in 0.5% BSA-DMEM for 1, 2 or 4 h at 37°C. Control group was treated with just the vehicle. In parallel, THP-1 monocytes (ATCC, Manassas, VA, USA) were labeled with Cytotrace™ Green CMFDA dye (29). Briefly, THP-1 cells were collected by centrifugation and resuspended at 5 × 10^5^ cells per tube in 500 μL of working dye solution prepared in serum-free DMEM medium. Dye solution freshly prepared was added to cells and incubated at 37°C in a light-proof box for 30 min. After incubation, the labeled THP-1 cells were washed twice with pre-warmed DMEM to remove residual unbound dye. Labeled THP-1 cells were then resuspended in pre-warmed growth medium, added to the LPS-stimulated HAECs monolayers to achieve a final concentration of 1 × 10^5^ cells per well, and co-incubated for 1 h at 37°C. Non-adherent labeled THP-1 cells were removed by washing the wells twice with pre-warmed DMEM. The remaining adherent cells were fixed with Image-iT™ Fixative Solutions in phosphate-buffered solution (Thermo Fisher Scientific) for 15 min at room temperature. Wells were then washed, covered with PBS, and imaged using a Leica DMI 400B fluorescence microscope (Leica Microsystems, Wetzlar, Germany). The number of adherent THP-1 cells per field was quantified using ImageJ software (NIH, Bethesda, MD, USA).

### 2.5 RNA isolation and gene expression analysis

HAECs were seeded in 6-well plates with 5 x 10^5^ cells per well and incubated at 37°C in a humidified atmosphere containing 5% CO_2_ overnight. The OCT group were pretreated at 2.5 µM in ECM overnight and the other two groups incubated with ECM for the same time. All the HAECs were washed with prewarmed PBS and then treated with LPS at 100 ng/mL in DMEM supplemented with 0.5% BSA for 4 h at 37°C. Total RNA was extracted with a Qiagen RNeasy^®^ Mini Kit, according to the manufacturer’s instructions. Total RNA concentration was measured, using a Nanodrop (Thermo Fisher Scientific). To quantitate expression of selected genes by RT-qPCR, complementary DNA was first synthesized from 1000 ng of total RNA, using iScript cDNA Synthesis Kit (Bio-Rad). mRNA levels of proinflammatory cytokines (IL-6, IL-8, TNF-α, IL-1β) and chemokine (MCP-1), adhesion molecules (E-selectin, P-selectin, ICAM-1,VCAM-1), and cytoskeleton protein (VE-Cadherin, β-Catenin, and ZO-1) were determined by RT-qPCR with TaqMan Gene Expression Assays and iTaq Universal Probes Supermix (Bio-Rad),. GAPDH or 18S were used as an internal control. The primer kits used for RT-qPCR can be found in **Table 2**.

**Table 2:** List of genes and primers used in RT-qPCR.

Protein	Gene name	Primers
IL-6	*IL6*	Hs00174131_m1
IL-8	*CXCL8*	Hs00174103_m1
IL-1β	*IL1B*	Hs01555410_m1
TNF-α	TNF	Hs00174128_m1
MCP-1	*CCL2*	Hs00234140_m1
VCAM-1	*VCAM1*	Hs01003372_m1
ICAM-1	*ICAM1*	Hs00164932_m1
P-selectin	*SELP*	Hs00927900_m1
E-selectin	*SELE*	Hs00174057_m1
VE-cadherin	*CDH5*	Hs00901463_m1
β-Catenin	*CTNNB1*	Hs00355045_m1
ZO-1	*TJP1*	Hs01551871_m1
Cortactin	*CTTN*	Hs01124232_m1
Vinculin	*VCL*	Hs00419715_m1
Talin	*TLN-1*	Hs00196775_m1
TLR-4	*TLR4*	Hs00152939_m1
TRAF-6	*TRAF6*	Hs00939742_g1
IRAK1	*IRAK1*	Hs00155570_m1
MYD88	*MYD88*	Hs00182082_m1
TIRAP	*TIRAP*	Hs01591921_g1
GAPDH	*GAPDH*	Hs02786624_g1
18S	*18S*	Hs99999901_s1

### 2.6 RNA-sequence analysis

Libraries were constructed from 500–800 ng of total RNA, using the Illumina’s TruSeq Stranded Total RNA kit with Ribo-Zero (Illumina, San Diego, CA, USA). The fragment size of RNAseq libraries was verified using the Agilent 2100 Bioanalyzer (Agilent Technologies, Santa Clara, CA, USA) and concentrations were determined using Qubit instrument (Thermo Fisher scientific). The libraries were loaded onto the Illumina NovaseqX (Illumina, San Diego, CA, USA) for 2 x 100 bp paired end read sequencing. The FASTQ files were generated using the bcl2fastq software. A total of ~2 million paired-end reads were generated with a base call quality of ≥Q30. Raw sequence reads in FASTQ format were trimmed using Trimmomatic (v0.39), and the filtered reads were aligned to the human reference genome (hg38) using STAR (v2.7.11b). Gene quantification was performed with RNA-Seq by Expectation-Maximization (RSEM), generating transcript-per-million (TPM) and fragments-per-kilobase-per-million mapped reads (FPKM). Differential gene expression (DGE) analysis was conducted using DESeq2. Pathway and functional enrichment analyses were performed using Ingenuity Pathway Analysis (IPA; QIAGEN, Venlo, Netherlands), using the MSigDB Hallmark gene sets to identify significantly enriched biological processes and canonical pathways.

### 2.7 Pro-inflammatory factors measurement by ELISA

The production of cytokines in cell culture supernatants of HAECs in response to LPS stimulation was quantified using enzyme-linked immunosorbent assay (ELISA). Interleukin-6 (IL-6), interleukin-8 (IL-8), and monocyte chemoattractant protein-1 (MCP-1) were measured using uncoated ELISA kits (Thermo Fisher Scientific). Tumor necrosis factor-alpha (TNF-α) and interleukin-1 beta (IL-1β) levels were assessed using precoated ELISA kits (R&D Systems, MN, USA). HAECs were seeded into 12-well plates at a density of 5.0 × 10^4^ cells/mL and incubated overnight at 37°C in a humidified atmosphere containing 5% CO_2_. The OCT group were pretreated at 2.5 µM in ECM overnight. All the cells were washed with prewarmed PBS and then treated with LPS at 100 ng/mL in DMEM supplemented with 0.5% BSA for 4 h at 37°C. The medium was collected and measured with the ELISA kits. The color signal was measured microplate reader (Bio-Tek Synergy H1, Vermont, USA).

### 2.8 Flow cytometry analysis of cell surface adhesion molecules

Subconfluent HAECs grown in 6-well plates after treatment with OCT were stimulated with 100 ng/mL LPS for 4 h at 37°C. Cells were then washed once with prewarmed phosphate-buffered saline (PBS), then detached using enzyme-free Cell Dissociation Buffer (Thermo Fisher Scientific). Detached cells were collected and washed with fluorescence-activated cell sorting (FACS) buffer, consisting of 5 mmol/L EDTA, 0.1% sodium azide (NaN_3_), and 1% fetal calf serum (Thermofisher Scientific) in Dulbecco’s PBS. Cells were incubated on ice for 1 h with fluorescently labeled monoclonal antibodies specific for human E-selectin, vascular cell adhesion molecule-1 (VCAM-1), and intercellular adhesion molecule-1 (ICAM-1) (Biolegend, USA) in a dark room. After antibody incubation, cells were washed twice with cold FACS buffer and analyzed for protein expression using flow cytometry in a BD LSRFortessa (BD Biosciences, Franklin Lakes, NJ, USA). Typically, the number of counted events was at least 10,000. Fluorescence intensities were recorded and analyzed to assess surface expression of adhesion molecules with FlowJo (BD Bioscience, OR, USA).

### 2.9 Endothelial permeability assay

To assess endothelial monolayer integrity and permeability, cells were grown in 24-well Transwell^®^ inserts with 0.4 μm pore polyester membranes pre-coated with Matrigel (Thermo Fisher Scientific) (30, 31). In brief, 2 × 10^5^ HAECs per 100 μL in ECM were carefully seeded into the upper chamber of each Transwell^®^ insert, with 1.5 mL of ECM in the lower chamber, and then incubated at 37°C overnight. For the OCT group, cells were treated with 2.5 μM OCT in ECM overnight. After cells were treated with 100 ng/mL LPS in DMEM with 0.5% BSA for 4 h, 150 μL of DMEM containing 10 μg/mL fluorescein isothiocyanate (FITC)-dextran (40 kDa) was added to the upper chamber of each insert. Inserts were transferred to a new 24-well plate receiver, with each lower chamber containing 1 mL of fresh DMEM. The plate was incubated at room temperature in the dark to allow diffusion of FITC-dextran through the cell monolayer. At 10, 20 and 30 min post-incubation, 100 μL of medium was collected from each receiver well and placed into a black opaque 96-well plate for fluorescence analysis. Fluorescence intensity of FITC-dextran in the collected samples was measured using a microplate spectrofluorometer (excitation at 485 nm; emission at 535 nm) (Bio-Tek Synergy H1, Vermont, USA).

A similar Transwell^®^ insert method was also used to measure the effects of OCT on LDL leakage after HAECs were treated by LPS. Fluorescently labeled LDL was prepared as previously described (32). Briefly, Human LDL (d: 1.019 – 1.064 g/mL) was isolated from plasma from healthy donors by preparative sequential KBr density ultracentrifugation (330,000 x g) followed by extensive dialysis at 4°C against PBS to remove KBr (28). Pure LDL (6 mg of protein) was then incubated with Alexa Fluor™ 568 carboxylic acid, succinyl ester (Molecular Probes, Eugene, OR) for 1 h at room temperature in the dark. Free, unbound dye was separated from labeled LDL by preparative fast protein liquid chromatography (FPLC) using a Hi-Trap Desalting column (GE Healthcare, Chicago, IL, USA). To assess HAECs integrity, 150 μL of DMEM containing 100 μg/mL Alexa Fluor™ 568-labelled LDL, was added to the upper chamber of each insert after LPS stimulation. The samples were collected as described above and measured in a microplate spectrofluorometer (excitation at 578 nm; emission at 602 nm).

### 2.10 Confocal fluorescence microscopy

For confocal imaging, 5 x 10^4^ HAECs were cultured in 35 mm Dish with 20 mm glass bottom Well (Cellvis, CA, USA). Following LPS treatment, cells were washed once with prewarmed PBS and fixed with Image-iT™ Fixative Solutions in PBS for 15 min at room temperature, then permeabilized with 0.1% Triton X-100 in PBS for 10 min. After blocking with 10% heated goat serum (Thermo Fisher Scientific) in PBS for 1 h, cells were incubated with primary antibodies targeting the proteins of interest (**Table 1**) overnight at 4°C. On the next day, cells were washed and incubated with species-appropriate fluorescent secondary antibodies (1:2000 dilution) and phalloidin labeling probes (Alexa Fluor™ 594 or Alexa Fluor™ 488, 1:400 dilution) for 1 h at room temperature in the light-proof dark box. Nuclei were counterstained with To-pro-3 (Thermo Fisher Scientific) for 10 min and examined using a Leica TCS SP5 confocal fluorescent microscope (Leica Microsystems, Wetzlar, Germany). Image acquisition settings were kept constant across all experimental conditions. Quantification of Fluorescence images were analyzed and quantified using ImageJ software.

### 2.11 Statistical analysis

Statistical analyses among the three groups were performed using GraphPad Prism version 9.5.1 (GraphPad Software Inc. La Jolla, CA, USA). Data are expressed as the mean ± SD, and analyzed by a one-way ANOVA followed by a Tukey *post hoc* test where appropriate. p < 0.05 was considered statistically significant. Each experiment was repeated at least 3 times under the same conditions.

## 3 Results

### 3.1 OCT inhibits the LPS-induced monocyte adhesion to HAECs in a dose- and time-dependent manner

**Figure 1:**
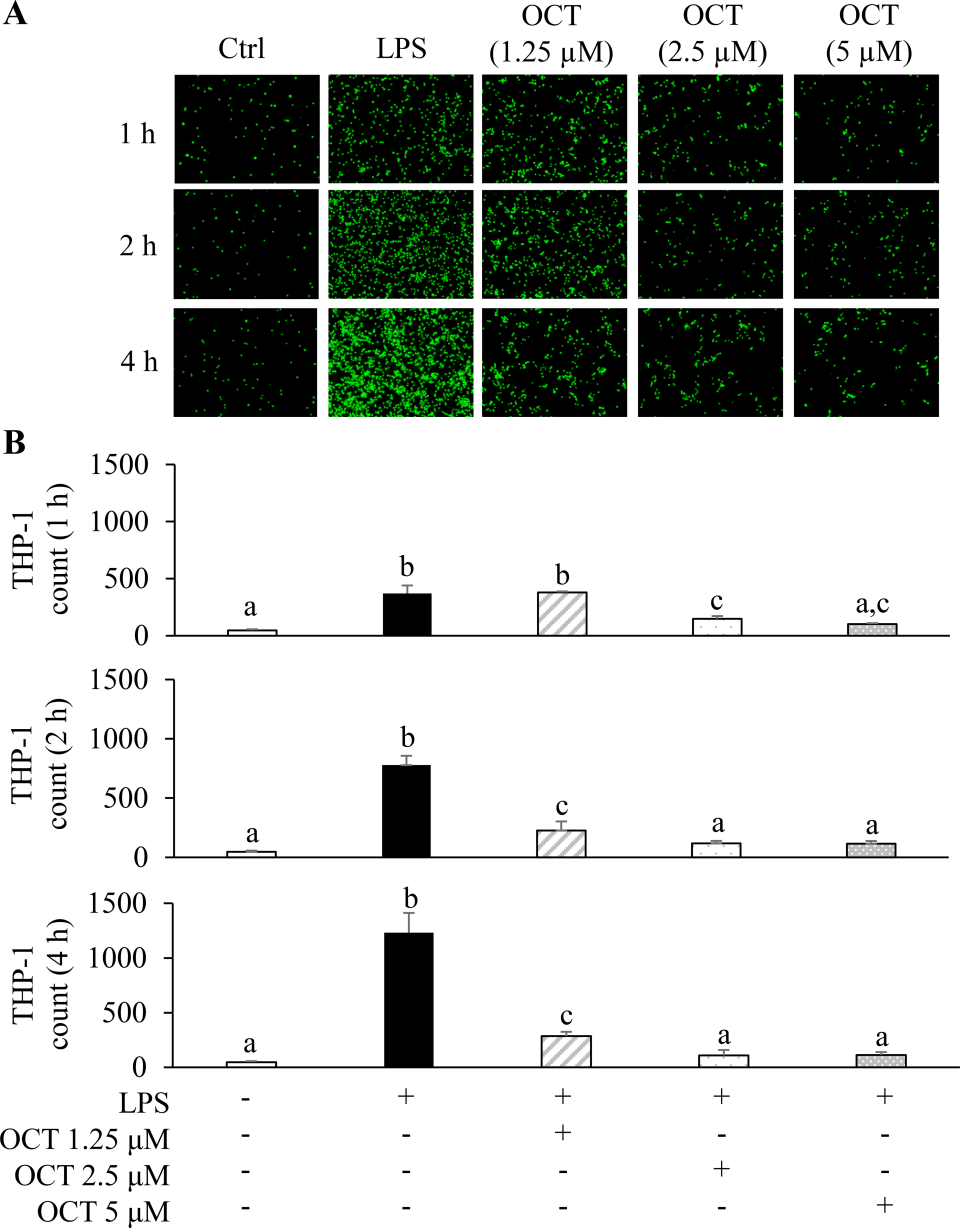
Concentration-dependent protective effect of OCT on LPS-induced monocyte adhesion to HAECs. **(A)** Time course of FITC-labeled fluorescent THP-1 cells observed under a fluorescent microscope; **(B)** Time course of FITC-labeled fluorescent THP-1 cell count quantification assessed by ImageJ software. HAECs co-cultured with FITC-labeled THP-1 cells were pretreated with OCT at 0, 1.25, 2.5, or 5 µM overnight, followed by LPS (100 ng/mL) stimulation for 1 h, 2 h, or 4 h. Control cells were treated with vehicle and without LPS stimulation. Data are mean ± SD (n=5) of three independent experiments. Letters (a, b, and c) above bars indicate statistically significant differences between group means. Different letters indicate significant differences (p < 0.05) between groups, according to one-way ANOVA followed by Tukey’s *post hoc* test, whereas the same letter means that there is no significant difference between groups according to one-way ANOVA followed by Tukey’s *post hoc* test. OCT, Octacosanol; HAECs, Human Aortic Endothelial Cells; LPS, lipopolysaccharide; FITC, Fluorescein isothiocyanate.

OCT treatment of HAECs at concentrations ranging from 0.5 to 10 μM did not result in any apparent cytotoxicity (**Supplementary Figure S1A**). We also tested different doses of LPS ranging from 10 to 10,000 ng/mL for 2, 4, and 6 h to evaluate HAEC viability. LPS at 100 ng/mL for 4 h only elicited a mild inflammatory response without inducing significant cytotoxicity (**Supplementary Figure S1B**). We, therefore, used 100 ng/mL of LPS for 4 h to examine the possible protective effect of OCT on HAECs in subsequent studies. In a time-course study, LPS stimulation led to a time-dependent increase in THP-1 cells adhesion to monolayer of HAECs (**Figure 1**). Adhesion of THP1 to HAECs stimulatedby LPS was significantly increased by 6.9-fold at 1 h, 15.6-fold at 2 h, and 25.3-fold at 4 h compared to unstimulated control group. However, when cells were pretreated with OCT overnight, LPS-induced THP-1 adhesion to HAECs was markedly reduced in a dose- and time-dependent manner (p < 0.05). 1.25 μM of OCT pretreatment reduced LPS-stimulated monocyte adhesion by 71% at 2 h (p < 0.05), and by 77% at 4 h (p < 0.05). 2,5 μM of OCT pretreatment reduced LPS-stimulated monocyte adhesion by 50% at 1 h (p < 0.05), 85% at 2 h (p<0.05), and 91% at 4 h (p < 0.05). 5 μM of OCT treatment significantly reduced LPS-stimulated monocyte adhesion by 72% at 1 h (p < 0.05), 85% at 2 h (p < 0.05), and 91% at 4 h (p < 0.05). Both 2.5 µM and 5 µM of OCT achieved maximal reduction of monocyte adhesion, at concentrations that blocked ~90% of THP-1 adhesion to HAECs after 4 h of LPS stimulation. We, therefore, selected 2.5 µM of OCT and 4 h treatment of LPS for subsequent experiments.

### 3.2 OCT reduces inflammation in LPS-treated HAECs

**Figure 2:**
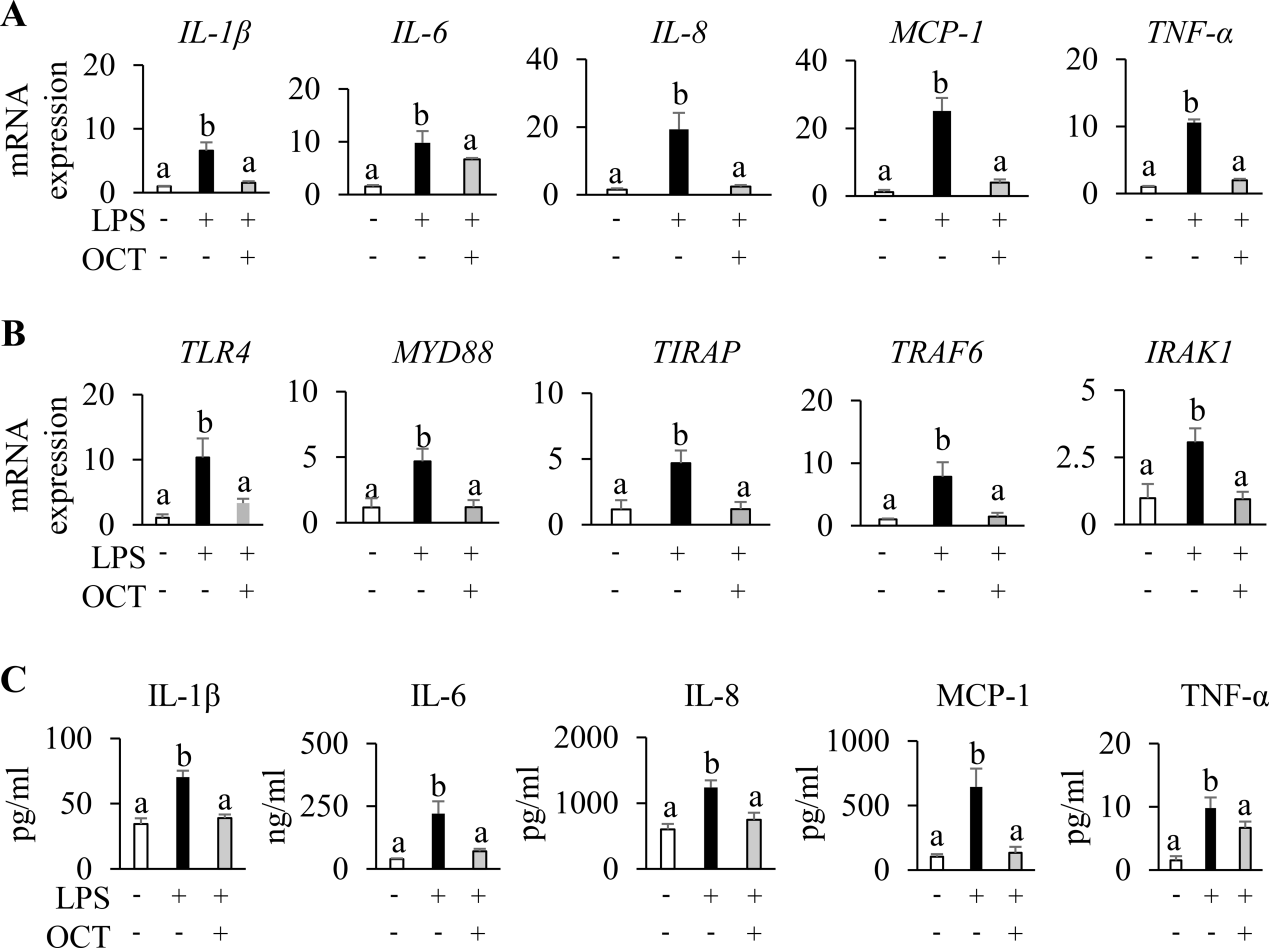
Protective effect of OCT on LPS-induced inflammatory response in HAECs. **(A)** Relative mRNA expression of *IL-1β*, *IL-6*, *IL-8*, *MCP*-1, and *TNFα* in HAECs; **(B)** Relative mRNA expression of *TLR4*, *MYD88*, *TIRAP*, *TRAF6*, and *IRAK1* in HAECs; **(C)** Protein concentrations of IL-1β, IL-6, IL-8, MCP-1, and *TNF-α* in the supernatant of HAECs. HAECs were pretreated with OCT at 2.5 µM overnight, followed by LPS (100 ng/mL) stimulation for 4 h. Control cells were treated with vehicle and without LPS stimulation. Data are mean ± SD (n=4) of three independent experiments. Letters (a, b) above bars indicate statistically significant differences between group means. Different letters indicate significant differences (p < 0.05) between groups, according to one-way ANOVA followed by Tukey’s *post hoc* test, whereas the same letter means that there is no significant difference between groups. OCT, Octacosanol; HAECs, Human Aortic Endothelial Cells; LPS, lipopolysaccharide; IL, Interleukin; MCP-1, Monocyte chemoattractant protein-1; TNFα, Tumor Necrosis Factor alpha.

To evaluate the anti-inflammatory effect of the OCT in HAECs, the mRNA expression and secreted pro-inflammatory cytokines were analyzed using RT-qPCR and ELISA, respectively. LPS treatment significantly increased mRNA levels of *IL-1β*, *IL-6*, *IL-8*, *MCP-1*, and *TNF-α* by 5 to 10-fold compared with the untreated control group (p < 0.05). However, OCT pretreatment reduced the mRNA expression of all these proinflammatory cytokines to near basal levels in HAECs stimulated with LPS (p < 0.05) (**Figure 2A**). ELISA analysis further confirmed the anti-inflammatory effect of OCT. LPS stimulation markedly increased the concentrations of IL-1β, IL-6, IL-8, MCP-1, and TNF-α in the cell culture supernatant compared with control (p < 0.05), whereas OCT pretreatment significantly decreased the release of these cytokines compared with LPS treatment alone (p < 0.05) (**Figure 2C**). To investigate the potential mechanism by which OCT influences LPS-induced inflammation, we also evaluated mRNA expression of several key genes in the TLR4-mediated NF-κB signaling pathway. LPS upregulated *TLR4*, *MYD88*, *TIRAP*, *TRAF6*, and *IRAK1* compared with control (p < 0.05), but this response was suppressed by OCT pretreatment (p < 0.05) (**Figure 2B**).

### 3.3 OCT inhibits LPS-induced expression of adhesion molecules in HAECs

**Figure 3:**
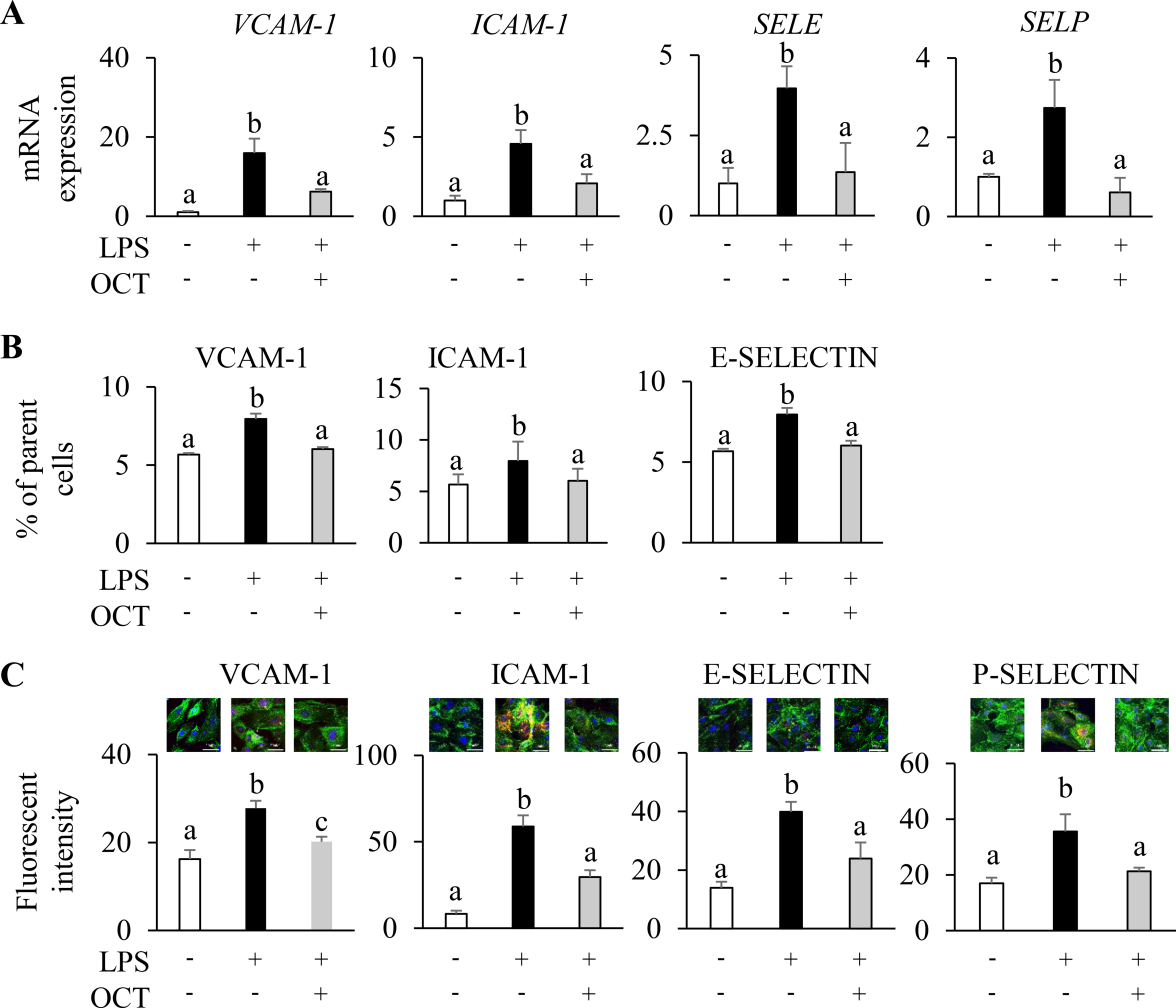
Inhibiting effect of OCT on LPS-induced overexpression of endothelial adhesion molecules in HAECs. **(A)** Relative mRNA expression of *VCAM-1*, *ICAM-1*, *SELE*, and *SELP* in HAECs; **(B)** Expression (% of parent cells) of VCAM-1, ICAM-1, and E-SELECTIN assessed by flow cytometry; **(C)** Representative confocal image (merged; 60X) and relative fluorescent intensity from HAECs. Stained HAECs showing green-fluorescent F-actin, blue-fluorescent nuclei were labeled against VCAM-1, ICAM-1, E-SELECTIN, or P-SELECTIN (red). Data are mean ± SD (n=4) of three independent experiments. HAECs were pretreated with OCT at 2.5 µM overnight, followed by LPS (100 ng/mL) stimulation for 4 h. Control cells were treated with vehicle and without LPS stimulation. Letters (a, b) above bars indicate statistically significant differences between group means. Different letters indicate significant differences (p < 0.05) between groups, according to one-way ANOVA followed by Tukey’s *post hoc* test, whereas the same letter means that there is no significant difference between groups. OCT, Octacosanol; HAECs, Human Aortic Endothelial Cells; LPS, lipopolysaccharide; VCAM-1, Vascular Cell Adhesion Molecule 1; ICAM-1, Intercellular Adhesion Molecule 1; SELE, E-selectin; SELP, P-selectin.

To explore possible mechanism underlying decreased monocyte adhesion to LPS-stimulated HAECs due to OCT pretreatment, we analyzed the expressions of several adhesion molecules. LPS stimulation significantly increased the mRNA levels of *VCAM-1*, *ICAM-1*, *SELE*, and *SELP* compared with the control group (p < 0.05), but OCT pretreatment significantly attenuated their upregulation from LPS treatment (p < 0.05) (**Figure 3A**). Flow cytometry analysis confirmed that LPS markedly increased the surface expression of VCAM-1, ICAM-1 and E-selectin (p < 0.05), whereas OCT-treated HAECs largely reversed the LPS-induced expression increase and maintained the protein levels of these adhesion proteins near control levels (p < 0.05) (**Figure 3B**). In addition, by confocal microscopy LPS treatment resulted in strong fluorescence signals corresponding to all four adhesion molecules around endothelial cells, as is evidenced by the significant shift by 3 to 10-fold increase in the mean fluorescent intensity relative to non-stimulated controls (p < 0.05). In contrast, OCT-pretreated cells displayed a markedly weaker fluorescence signal for these adhesion proteins (p < 0.05) (**Figure 3C**; **Supplementary Figures S2-S5**).

### 3.4 OCT protects against LPS-induced endothelial cell barrier disruption

**Figure 4:**
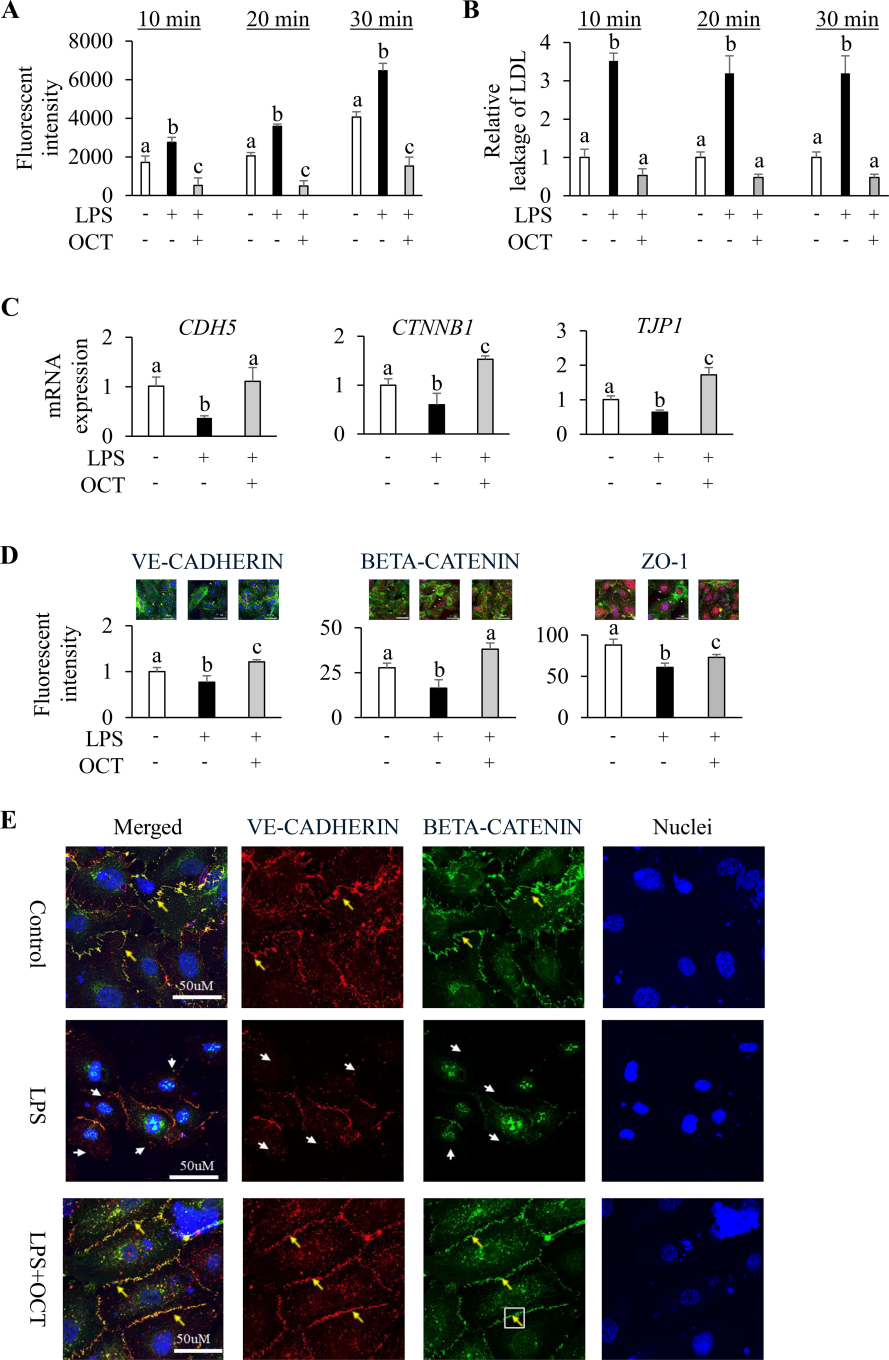
Protective effect of OCT on LPS-induced endothelial cell barrier disruption and permeability. **(A)** Fluorescent intensity of FITC-Dextran measured at the bottom compartments of transwells through HAECs monolayer after a 10, 20, or 30 min incubation time; **(B)** Relative fluorescent intensity of Alexa Fluor™ 568 -labeled LDL measured at the bottom compartments of transwells through HAECs monolayer after a 10, 20, or 30 min incubation time; **(C)** Relative mRNA expression of *CDH5*, *CTNNB1*, and *TJP1* in HAECs; **(D)** Representative confocal image (merged; 60X) and relative fluorescent intensity from HAECs. Stained HAECs showing green-fluorescent F-actin, blue-fluorescent nuclei were labeled against VE-CADHERIN, BETA-CATENIN, or ZO-1 (red). Yellow arrows stand for VE- CADHERIN, BETA-CATENIN or ZO-1 stained intact around cell border, and white arrows stand for defects of the targeted protein around the cell edges; **(E)** Confocal image (60X) of HAECs. Stained HAECs showing blue-fluorescent nuclei were labeled against VE-CADHERIN (red) and BETA-CATENIN (green). Yellow arrows stand for VE-CADHERIN and BETA-CATENIN colocalized in cell-cell junction, and white arrows stand for defects of both protein stained. Data are mean ± SD (n=4) of three independent experiments. HAECs were pretreated with OCT at 2.5 µM overnight, followed by LPS (100 ng/mL) stimulation for 4 h. Control cells were treated with vehicle and without LPS stimulation. Letters (a, b, and c) above bars indicate statistically significant differences between group means. Different letters indicate significant differences (p < 0.05) between groups, according to one-way ANOVA followed by Tukey’s *post hoc* test, whereas the same letter means that there is no significant difference between groups. OCT, Octacosanol; HAECs, Human Aortic Endothelial Cells; LPS, lipopolysaccharide; CDH5, VE-Cadherin; CTNNB1, β-Catenin; TJP1, Tight junction protein 1.

To examine the protective effect of the OCT on LPS-induced endothelial monolayer barrier disruption, we performed two different assays of endothelial function. LPS stimulation significantly increased the passage of FITC-dextran across the endothelial monolayer at all measured time points compared with control (p < 0.05). Pretreatment with the OCT, however, markedly reduced FITC-dextran infiltration through the monolayer HAECs at each time point compared with LPS treatment alone (p < 0.05) (**Figure 4A**). In a fluorescent labelled LDL leakage assay, LPS stimulation enhanced the leakage of LDL particles across the endothelial monolayer by approximately 3-fold compared with control (p < 0.05). OCT pretreatment, however, significantly attenuated LPS-induced LDL leakage at all the tested time points (p < 0.05), thus maintaining the endotheial barrier function similar to the control cells (**Figure 4B**).

To further explore the influence of OCT on cytoskeletal and junctional proteins in HAECs under inflammatory conditions, we investigated the changes of endothelial adherens and tight junctions. RT-qPCR analysis showed that LPS downregulated the mRNA expression of junctional proteins, such as *CDH5*, *CTNNB1*, and *TJP1*, compared with control (p < 0.05) (**Figure 4C**), but the mRNA expressions of these genes was preserved after OCT pretreatment. Confocal imaging analysis further corroborated these findings. LPS-treated HAECs exhibited remarkably reduced fluorescence intensities for VE-cadherin, β-catenin, and ZO-1 compared with control (p < 0.05) (**Figure 4D**; **Supplementary Figures S6-S8**). LPS stimulation also resulted in ruptures around the cell border and the fluorescently labeled proteins were often redistributed toward the perinuclear region. In contrast, pretreatment with OCT significantly counteracted these LPS-induced alterations, preserving cytoskeletal organization and maintaining junctional protein expression near basal levels. Notably, F-actin fibers remained well organized and smoothly extended in a similar pattern to those of control cells. In addition, co-localization analysis of VE-cadherin and β-catenin further showed that LPS treatment caused substantial rupture of cell–cell contacts, whereas OCT-treated cells retained intact and continuous cell borders after LPS stimulation (**Figure 4E**).

### 3.5 OCT inhibits LPS-induced focal adhesion in HAECs

**Figure 5:**
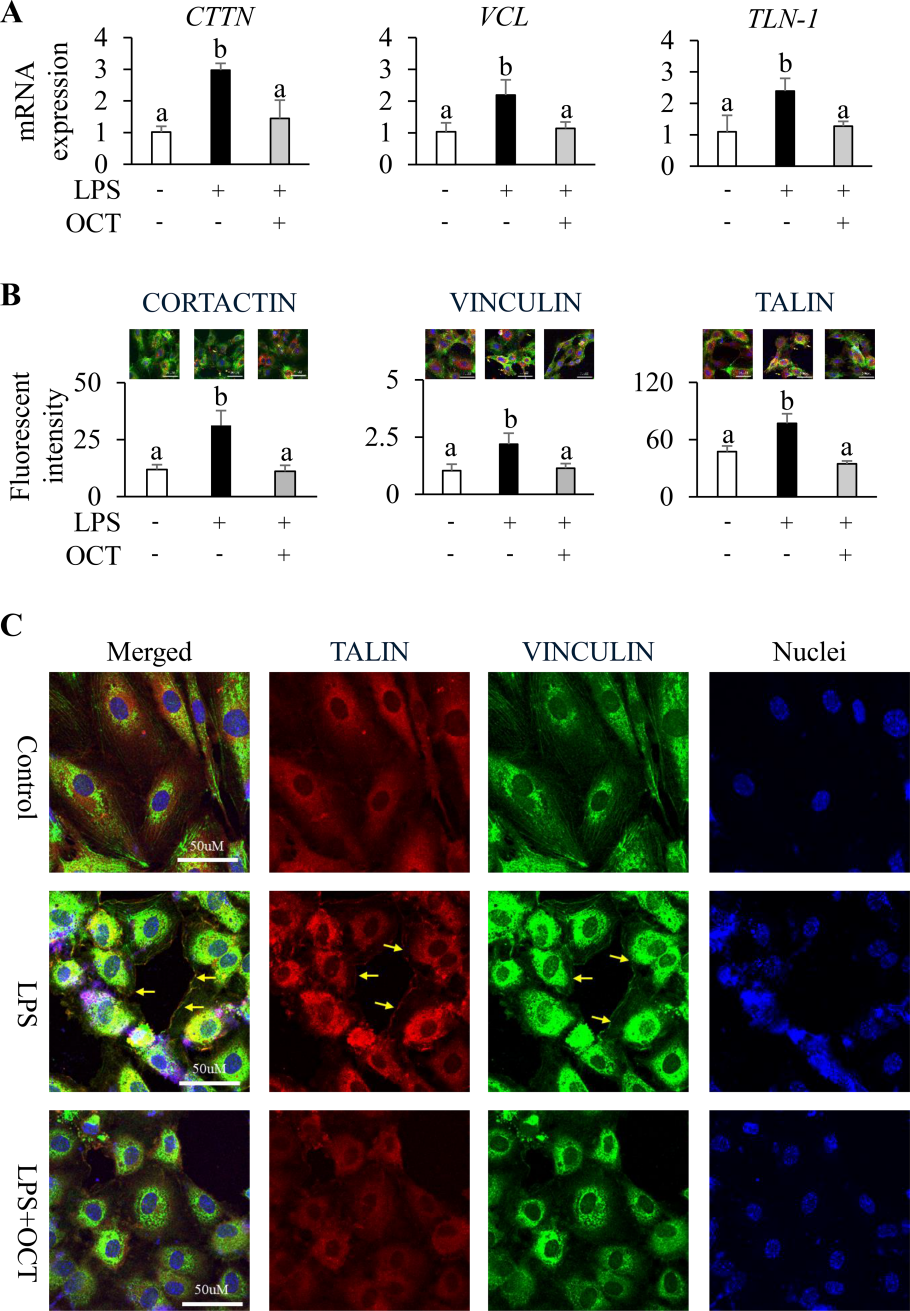
Protective effect of OCT on LPS-induced alternation of junction-associated protein expressions. **(A)** Relative mRNA expression of *CTTN*, *VCL*, and *TLN-1* in HAECs; **(B)** Representative confocal image (merged; 60X) and relative fluorescent intensity from HAECs. Stained HAEC showing green-fluorescent F-actin, blue-fluorescent nuclei were labeled against CORTACTIN (red) (Yellow arrows stand for CORTACTIN aggregated in cell protrusion appears lace-like border), VINCULIN (red) (Yellow arrows stand for VINCULIN enriched in cell lamellipodia and showed match-head like distribution), or TALIN (red) (Yellow arrows stand for TALIN enriched in cell lamellipodia and showed match-head like distribution); **(C)** Confocal image (60X) of HAECs. Stained HAECs showing blue-fluorescent nuclei were labeled against TALIN (red) and VINCULIN (green). Yellow arrows stand for TALIN and VINCULIN colocalized in cell protruded borders under LPS treatment. Data are mean ± SD (n=4) of three independent experiments. HAECs were pretreated with OCT at 2.5 µM overnight, followed by LPS (100 ng/mL) stimulation for 4 h. Control cells were treated with vehicle and without LPS stimulation. Letters (a and b) above bars indicate statistically significant differences between group means. Different letters indicate significant differences (p < 0.05) between groups, according to one-way ANOVA followed by Tukey’s *post hoc* test, whereas the same letter means that there is no significant difference between groups. OCT, Octacosanol; HAECs, Human Aortic Endothelial Cells; LPS, lipopolysaccharide; CTTN, Cortactin; VCL, Vinculin.

Upon LPS stimulation, mRNA expressions of *CTTN*, *VCL* and *TALIN* were significantly upregulated compared with control (p < 0.05), but OCT pretreatment effectively suppressed their expression to levels observed in control cells (**Figure 5A**). Immunofluorescent imaging revealed that OCT pretreatment consistently decreased protein levels of CORTACIN, VINCULIN, and TALIN, and prevented their redistribution compared with LPS group (p < 0.05) (**Figure 5B**). In normal control cells, cortactin was primarily distributed evenly around nuclei in the perinuclear cytoplasm. Upon LPS stimulation, however, cortactin became strongly polarized toward the cell periphery, accumulating within thin, finger-like protrusions that extended into the extracellular matrix. Most cortactin colocalized with actin, redistributed along the cell border, and contributed to the formation of protrusive structures that guided directional cell migration (**Supplementary Figure S9**). Following LPS stimulation, both vinculin and talin exhibited dense clustering along the cell periphery, with especially sharp enrichment at protrusive edges where they formed distinct, match-head–like focal accumulations (**Supplementary Figures S10, S11**). Further analysis using co-localization of Talin and Vinculin immunofluorescence labeling revealed that talin and vinculin co-localized at the tips of protruding actin filaments within lamellipodial and filopodial extensions at the periphery of LPS-treated endothelial cells. In contrast, OCT-pretreated cells exhibited markedly reduced talin and vinculin fluorescence intensities, fewer membrane protrusions, and a more stable cellular morphology resembling control cells (**Figure 5C**).

### 3.6 OCT-induced transcriptomic profile alterations in LPS-treated HAECs

**Figure 6:**
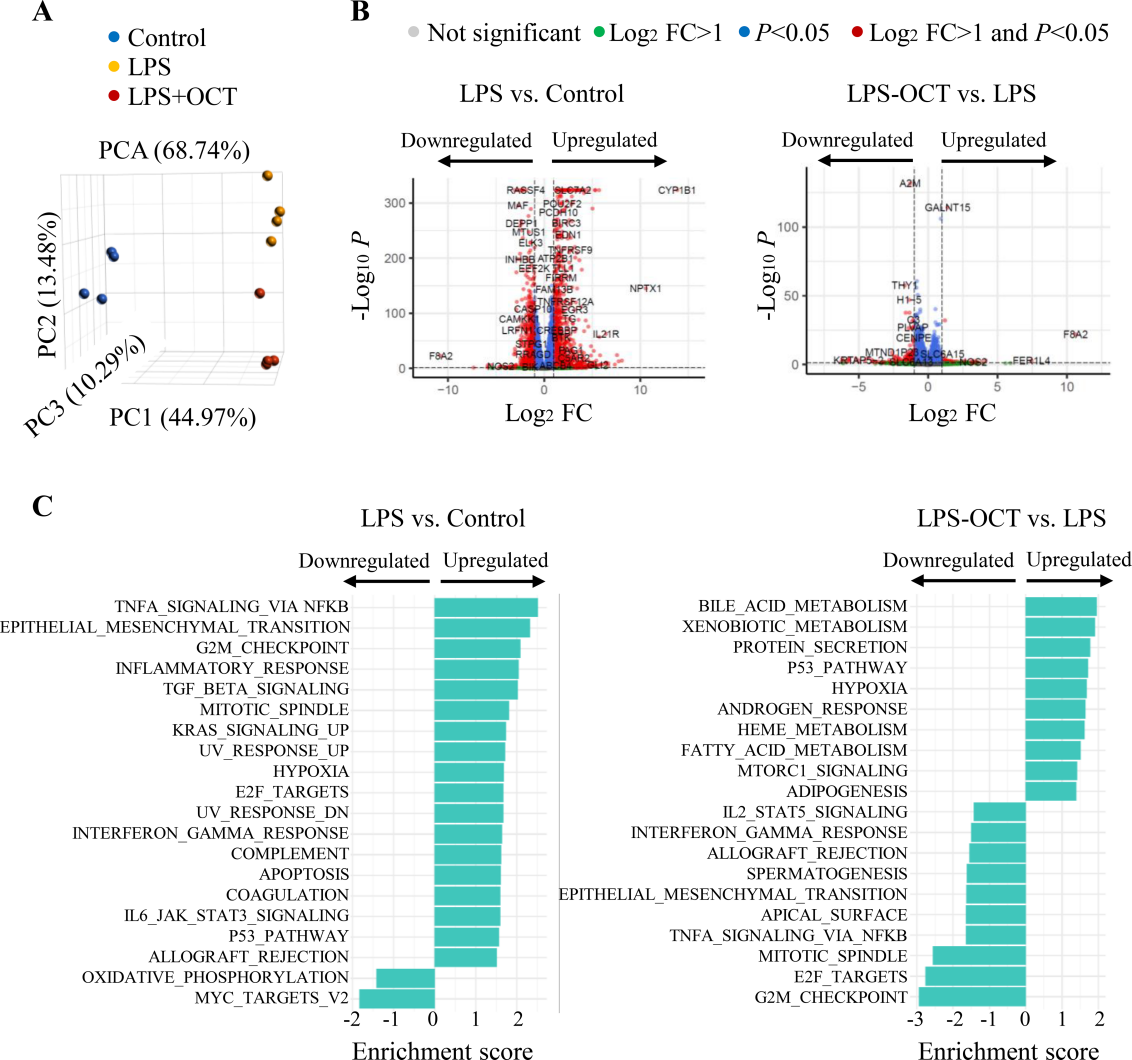
Transcriptomic profiling in response to OCT in LPS-stimulated HAECs. **(A)** Principal component analysis (PCA) showing overall separation of LPS and LPS-OCT treated HAECs from untreated HAECs (Control). Each dot represents RNA-Seq data from biologic triplicates; **(B)** Volcano plot showing the relative abundances of transcripts of LPS treated HAECs compared with untreated HAECs (Control) (left panel) and those of transcripts of LPS-OCT treated HAECs compared with LPS-treated HAECs (right panel). Transcripts with an FC value > 1.2 and an adjusted p-value < 0.05 were considered to be differentially expressed between the compared groups; **(C)** Pathway enrichment analysis showing the top 20 enriched IPA pathways. OCT, Octacosanol; HAECs, Human Aortic Endothelial Cells; LPS, lipopolysaccharide; FC, fold change; IPA, Ingenuity Pathway Analysis.

To explore the molecular mechanism underlying the cytoprotective effect of OCT in LPS-treated HAECs, RNA-seq analysis was conducted to compare the gene expression profiles of LPS-treated HAECs with or without OCT pretreatment. Principle Component Analysis (PCA) were employed to discern the impact of OCT treatment on gene expression levels. PCA analysis (**Figure 6A**) revealed three well-distinguished groups, indicating different gene expression patterns between the control, LPS and LPS plus OCT groups. Volcano plots (**Figure 6B**) of differentially expressed genes (DEGs) are shown for the LPS treatment compared with control (**Figure 6B**, left panel) and LPS plus OCT treatment compared with LPS alone (**Figure 6B**, right panel), respectively. LPS treatment resulted in 5117 DEGs, with 3010 genes up-regulated and 2107 genes down-regulated. In LPS-stimulated cells, OCT treatment resulted in 497 DEGs, with 117 genes up-regulated and 380 gene down-regulated. Pathway enrichment analysis further clarified the functional roles of DEGs (**Figure 6C**). LPS stimulation upregulated pathways related to cytokine and chemokine activity, several basic metabolic processes, and cell integrity pathways, indicating that LPS, at least in part, damages endothelial cells by triggering inflammation (**Figure 6C**, left panel). OCT treatment, however, reversed most of the DEGs induced by LPS in endothelial cells, primarily by downregulating inflammatory responses, including TNFα/NF-κB signaling, interferon-γ signaling, and IL2-STAT5 signaling pathways. OCT pretreatment also upregulated several pathways involved in lipid metabolism, including bile acid metabolism and fatty acid metabolism (**Figure 6C**, right panel).

## 4 Discussion

LPS is widely used *in vitro* to induce pro-inflammatory pathways in cultured endothelial cells (26, 27), but it may also be an important physiologic trigger of inflammation in humans (33, 34). We used a relatively low dose of LPS in our studies to simulate the type of chronic low-grade endothelial inflammation that occurs with atherosclerosis. Under these conditions, we reported for the first time that OCT protects HAECs from LPS-induced damage by decreasing proinflammatory cytokines and chemokines, by down-regulating the expression of several adhesion molecules, and by enhancing HAECs membrane integrity. These multiple independent effects of OCT could help explain its previously observed anti-inflammatory properties in animal models (25, 35).

As observed in previous studies (26, 36), LPS induced the expression of ICAM-1, VCAM-1 and pro-inflammatory cytokines in HAECs via activation of TLR4. Many of these effects could be reversed by OCT in a time- and dose-dependent manner, leading to reduced endothelial-leukocyte interactions. OCT treatment also reduced the expression of several endothelial adhesion molecules, including VCAM-1, ICAM-1, E-selectin, and P-selectin. These molecules are essential mediators of leukocyte recruitment, adhesion and vascular infiltration during inflammation (3). Gene expression analysis revealed that OCT downregulated transcript levels of several key components of the canonical TLR4– MyD88 cascade signaling pathway, including *TLR4*, *MyD88*, *TIRAP*, *IRAK1*, and *TRAF6* (37). TLR4 mediates the recruitment of intracellular adaptor molecules, such as TIRAP and MyD88. Downstream of MyD88, IL-1R-associated kinases (IRAKs) are also recruited, which leads to the activation of the E3 ubiquitin ligase TRAF6, culminating in the activation of NF-κB and MAPK through MyD88 dependent and independent pathways (37, 38). By reducing transcription of these upstream adaptors and kinases, OCT appears to interfere with the early stages of LPS signaling, thereby dampening the downstream inflammatory cascade. Similar inhibitory effects on TLR4/MyD88 expression and downstream MAPK/NF-κB activation have been observed with other small molecules, such as 4-octyl itaconate in endothelial cells (39, 40).

Pathway enrichment analysis further confirmed that OCT inhibited multiple pro-inflammatory signaling pathways, including IL-2/STAT5, interferon-gamma (IFN-γ) response, and TNF-α signaling via NF-κB. TNF-α/NF-κB signaling drives transcription of adhesion molecules and cytokines, while IL- 2/STAT5 and IFN-γ pathways amplify leukocyte activation and endothelial adhesion (37, 41, 42). The coordinated inhibition of these multiple pathways by OCT aligns with our functional observations, such as reduced cytokine release, suppressed adhesion molecule expression, and decreased monocyte adhesion. By blocking LPS-induced monocyte adhesion to HAECs, OCT can possibly prevent excessive or prolonged inflammatory responses, which are typical of chronic inflammatory conditions like atherosclerosis (43, 44). By simultaneously targeting both upstream TLR4 signaling and functional endpoints of inflammation, OCT may break the self-amplifying loop of inflammation, in which activated endothelial cells recruit immune cells that, in turn, release more inflammatory signals and further activate the endothelium, leukocyte recruitment, and vascular damage (45, 46).

Endothelial cell membrane permeability depends on precisely regulated barrier function within the vasculature to maintain physiological stability and facilitate essential substance transport. Endothelial cells achieve this through specialized adherens and tight junction protein complexes, which govern paracellular permeability across vascular beds (6, 47). It has been observed that LDL and oxidized LDL contribute to the atherosclerotic plaque formation and progression through several mechanisms, including the induction of endothelial cell activation and dysfunction, macrophage foam cell formation, and smooth muscle cell migration and proliferation (5, 8). In this study, we demonstrated that OCT preserved endothelial barrier integrity by affecting cell junctions and attenuating LPS-induced cell membrane LDL leakage. This mechanism supports *in vivo* findings where OCT prevented plaque formation in animal models, as endothelial dysfunction is a major, early driver of atherosclerosis (45, 48, 49). LPS not only decreased the expression of junction proteins, but it also disrupted both adherens and tight junctions (50). Notably, our immunofluorescence staining of VE-cadherin and β-catenin showed that they co-localized at cell periphery with consistent formation of intercellular gap in control and OCT treated cells, but large marginal defects in LPS treated cells. Previous studies have confirmed that inflammatory mediators, such as TNF-α, IL-1β and IL-6, promote VE-cadherin degradation and internalization by inducing the phosphorylation of specific tyrosine or serine residues (51, 52). Phosphorylation of VE-cadherin then disrupts the binding of p120 and β-catenin to induce internalization, resulting in the rupture of adherens junction (7, 53). Cytokines also perturb tight junctions by decreasing ZO-1 localization at the membrane, disrupting its linkage to the actin cytoskeleton, and increasing paracellular permeability by activation of MAPKs pathway (54–56). LPS transforms the apical surface from a quiescent, protective interface into an activated, highly adhesive, and leaky surface by different pathways, which then could enhance LDL vessel wall penetration, a critical step in atherosclerosis development (57). Our results show that OCT treatment can reverse many of the adverse effects of LPS on cell-cell junctions and endothelial permeability.

In addition to the protective effects on adherens and tight junctions, OCT also prevented LPS-induced focal adhesion formation of HAECs. The talin-vinculin axis is a key mechanosensing component of cellular focal adhesions (58, 59). Immunofluorescent analysis demonstrated that LPS stimulation enhances endothelial cytoskeletal remodeling by upregulating and activating the focal adhesion proteins, including talin and vinculin, which function as a tightly coupled mechanotransduction pair. Vinculin’s head domain binds talin, while its tail domain associates with F-actin, forming a mechanical clutch that reinforces focal adhesions and amplifies cytoskeletal tension (59, 60). Vinculin and the disruption of the endothelial cytoskeleton is a gateway for causing endothelial injury and inflammation (61). Our confocal imaging revealed that talin and vinculin concentrated and co-localized along the lace-like folded edges of LPS-treated HAECs, forming protrusive, sticky finger–like extensions into the extracellular matrix that was reported previously (62). OCT effectively reduced the recruitment of these proteins, thereby preventing endothelial elongation, cytoskeletal deformation, and cell migration. Additionally, OCT inhibited the LPS-induced increase in expression and distribution of cortactin in HAECs, which enhance Arp2/3-mediated actin branching, membrane protrusions, lammellipodia formation and the epithelial–mesenchymal transition (63–65).

Besides the anti-inflammatory effects, our pathway enrichment analysis also suggested that OCT upregulated several genes involved in basic metabolic pathways like bile acid metabolism and fatty acid metabolism, as previously described (22, 23, 45). Additional studies are, therefore, needed to fully understand the detailed molecular action of OCT. It is also important to note that OCT undergoes metabolism by β-oxidation and can be rapidly converted into octacosanoic acid and many other types of fatty acids (66, 67). Thus, some of the biological effects of OCT could be mediated by one of its many metabolites. In addition, longer LPS exposure (>24 h) can induce stress-induced premature senescence, which should be investigated future studies to evaluate both the acute and sustained anti-inflammatory effects of OCT (68, 69).

In conclusion, our findings identify OCT as a promising nutraceutical with endothelial-protective effects capable of reducing LPS-induced inflammatory activation, possibly by inhibiting the TLR4/NF-κB signaling pathway, while reinforcing cell–cell junctions and preserving barrier integrity. These results provide support for future research studies on the possible therapeutic value of OCT for conditions associated with vascular inflammation.

## Data Availability

The datasets presented in this study can be found in online repositories. The names of the repository/repositories and accession number(s) can be found in the article/**Supplementary Material**.

## Author disclaimer

The contributions of the NIH author(s) are considered Works of the United States Government. The findings and conclusions presented in this paper are those of the author(s) and do not necessarily reflect the views of the NIH or the U.S. Department of Health and Human Services.
