# Supplementary material for: Cytoprotective role of octacosanol in lipopolysaccharide-induced inflammation
Source: Front Immunol. 2026 Feb 11;17:1770191. doi: 10.3389/fimmu.2026.1770191 (PMC12932935; doi:10.3389/fimmu.2026.1770191)

Supplemental Materials

Supplemental Figure 1. Effect of OCT and LPS at different concentrations on the viability of HAECs. **(A)** Relative cell viability after treatment of OCT at 0 (control), 0.1625, 0.325, 0.625 μM, 1.25, 2.5, 5, or 10 μM for 48 h; **(B)** Relative cell viability after treatment of LPS at 0 (control), 10, 100, 500, 1000, or 10000 ng/mL for 2, 4, or 6 h. Data are mean ± SD (n=5) of three independent experiments. *p < 0.05, **p < 0.01, and ***p < 0.002 compared with control. HAECs were pretreated with OCT or LPS at designed concentrated for designed incubation time. Control cells were treated with vehicle. OCT: Octacosanol, HAECs: Human Aortic Endothelial Cells, LPS: lipopolysaccharide.

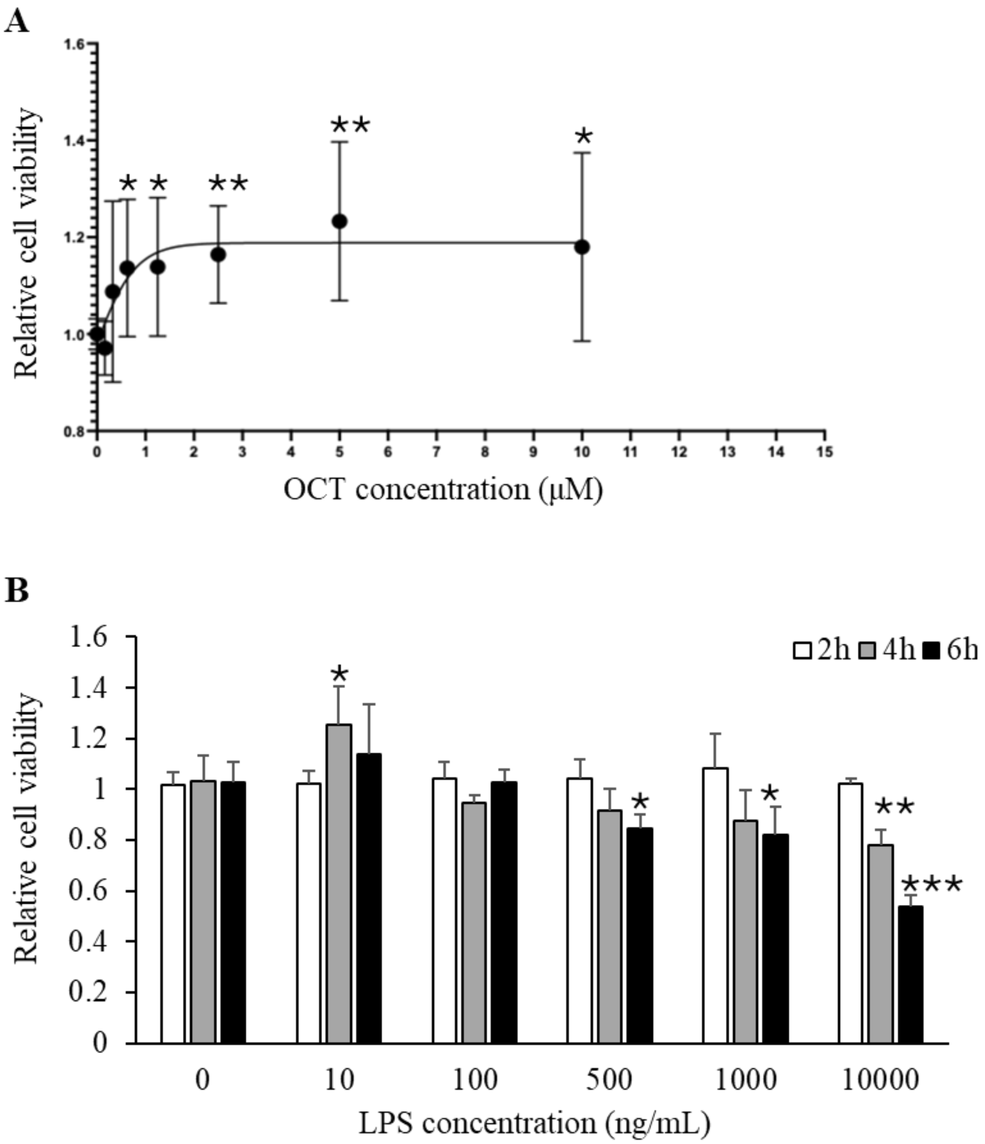

Supplemental Figure 2. Representative confocal image (60X) from HAECs. Panels from left: Merged HAECs image, HAECs labeled against VCAM-1 (red), F-actin stained HAEC labeling sub-cellular organelles (green), and To-pro-3 stained nuclei (blue). Upper panel (Ctrl): Control HAECs treated with vehicle and without LPS stimulation; middle panel (LPS): HAECs pretreated with vehicle overnight, followed by LPS (100 ng/mL) stimulation for 4 h. Bottom panel (LPS+OCT): HAECs pretreated with OCT at 2.5 μM overnight, followed by LPS (100 ng/mL) stimulation for 4 h. Yellow arrows stand for VCAM-1 under LPS treatment. Ctrl: Control, VCAM-1: Vascular Cell Adhesion Molecule 1.

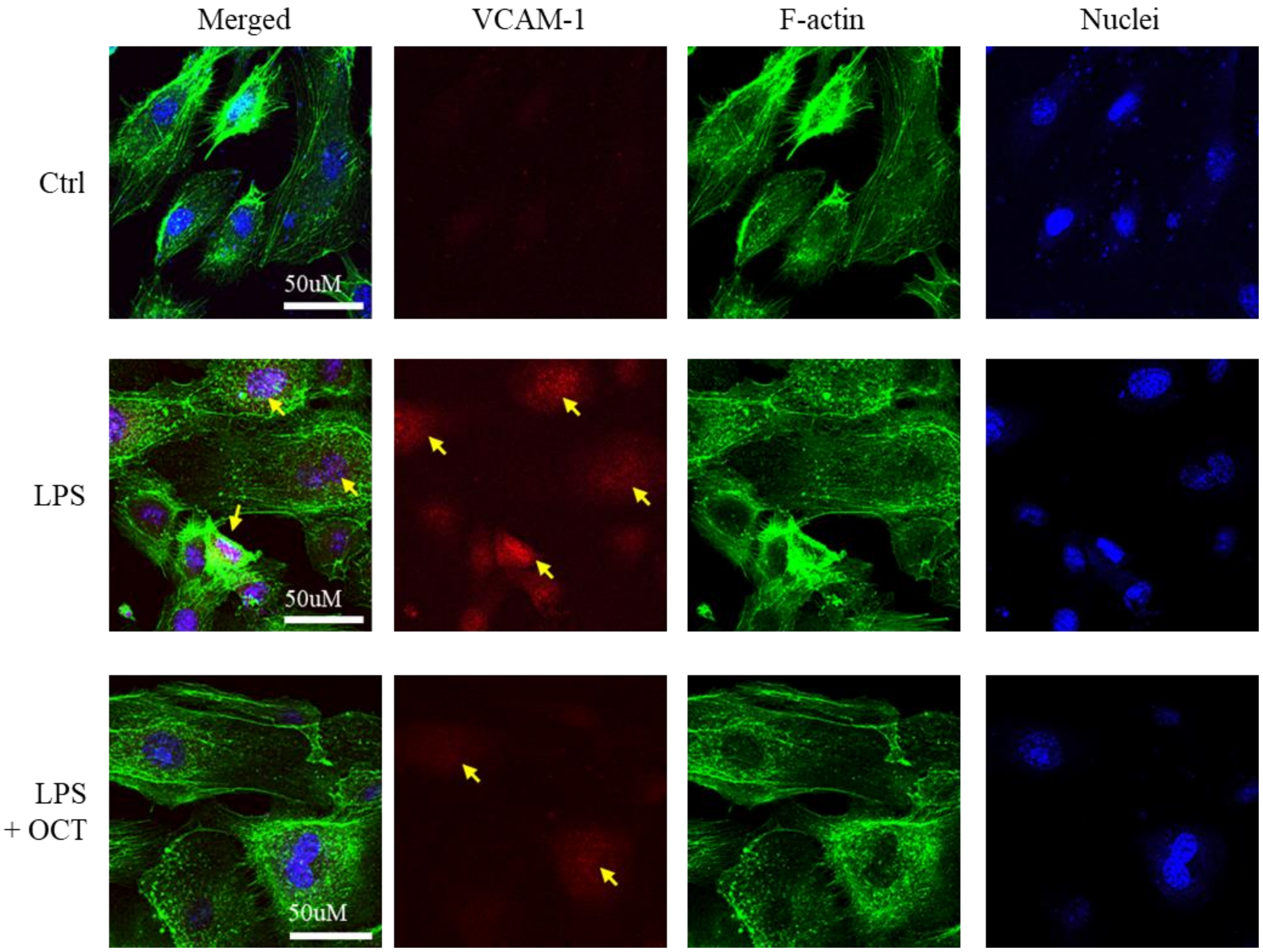

Supplemental Figure 3. Representative confocal image (60X) from HAECs. Panels from left: Merged HAECs image, HAECs labeled against ICAM-1 (red), F-actin stained HAEC labeling sub-cellular organelles (green), and To-pro-3stained nuclei (blue). Upper panel (Ctrl): Control HAECs treated with vehicle and without LPS stimulation; middle panel (LPS): HAEC pretreated with vehicle overnight, followed by LPS (100 ng/mL) stimulation for 4 h; bottom panel (LPS+OCT): HAECs pretreated with OCT at 2.5 µM overnight, followed by LPS (100 ng/mL) stimulation for 4 h. Yellow arrows stand for ICAM-1 aggregated under LPS treatment. Ctrl: Control, ICAM-1: Intercellular Adhesion Molecule 1.

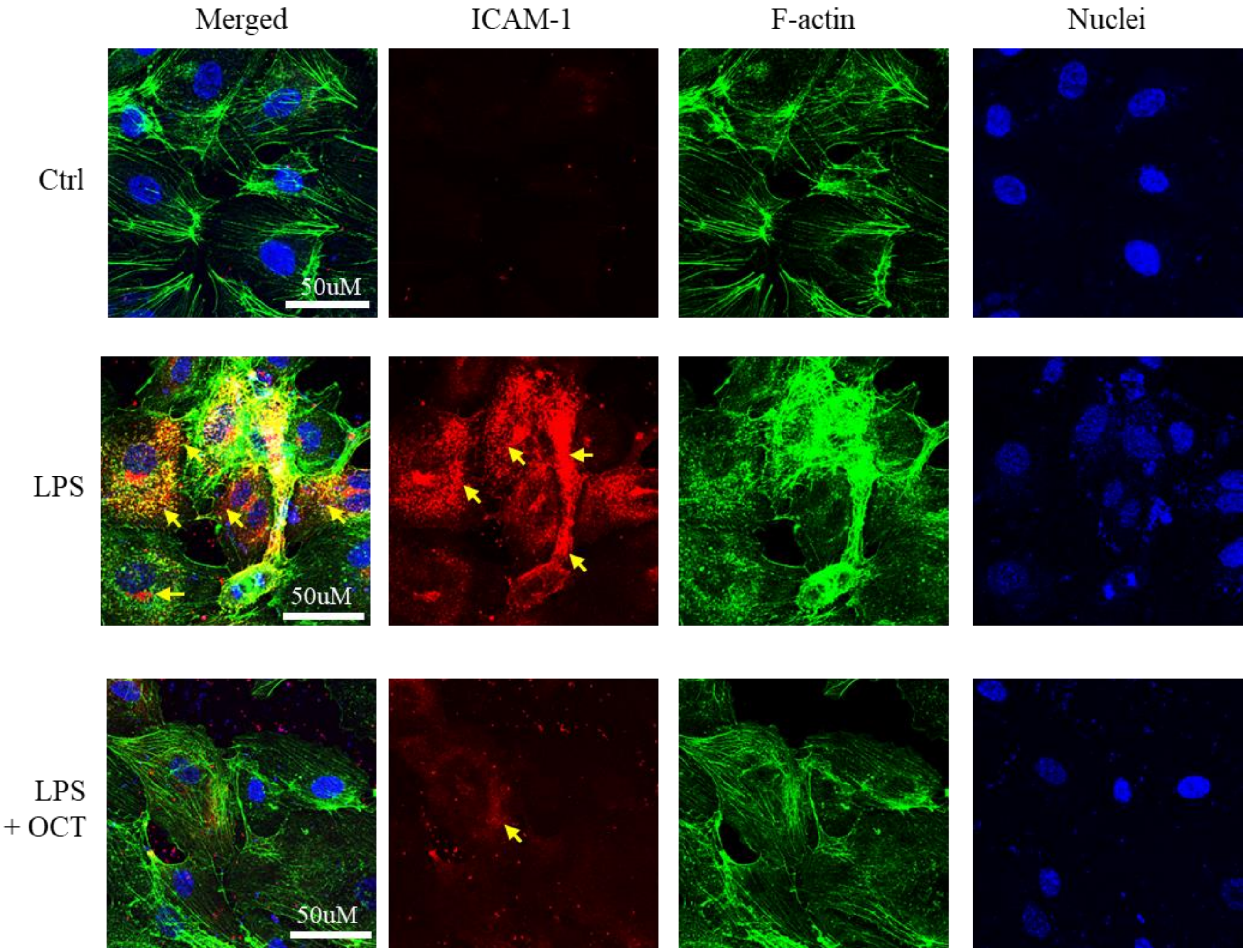

Supplemental Figure 4. Representative confocal image (60X) from HAECs. Panels from left: Merged HAECs image, HAEC labeled against E-SELECTIN (red), F-actin stained HAEC labeling sub-cellular organelles (green), and To-pro-3 stained nuclei (blue). Upper panel (Ctrl): Control HAECs treated with vehicle and without LPS stimulation; middle panel (LPS): HAECs pretreated with vehicle overnight, followed by LPS (100 ng/mL) stimulation for 4 h; bottom panel (LPS+OCT): HAECs pretreated with OCT at 2.5 μM overnight, followed by LPS (100 ng/mL) stimulation for 4 h. Yellow arrows stand for E-SELECTIN cluster under LPS treatment. Ctrl: Control.

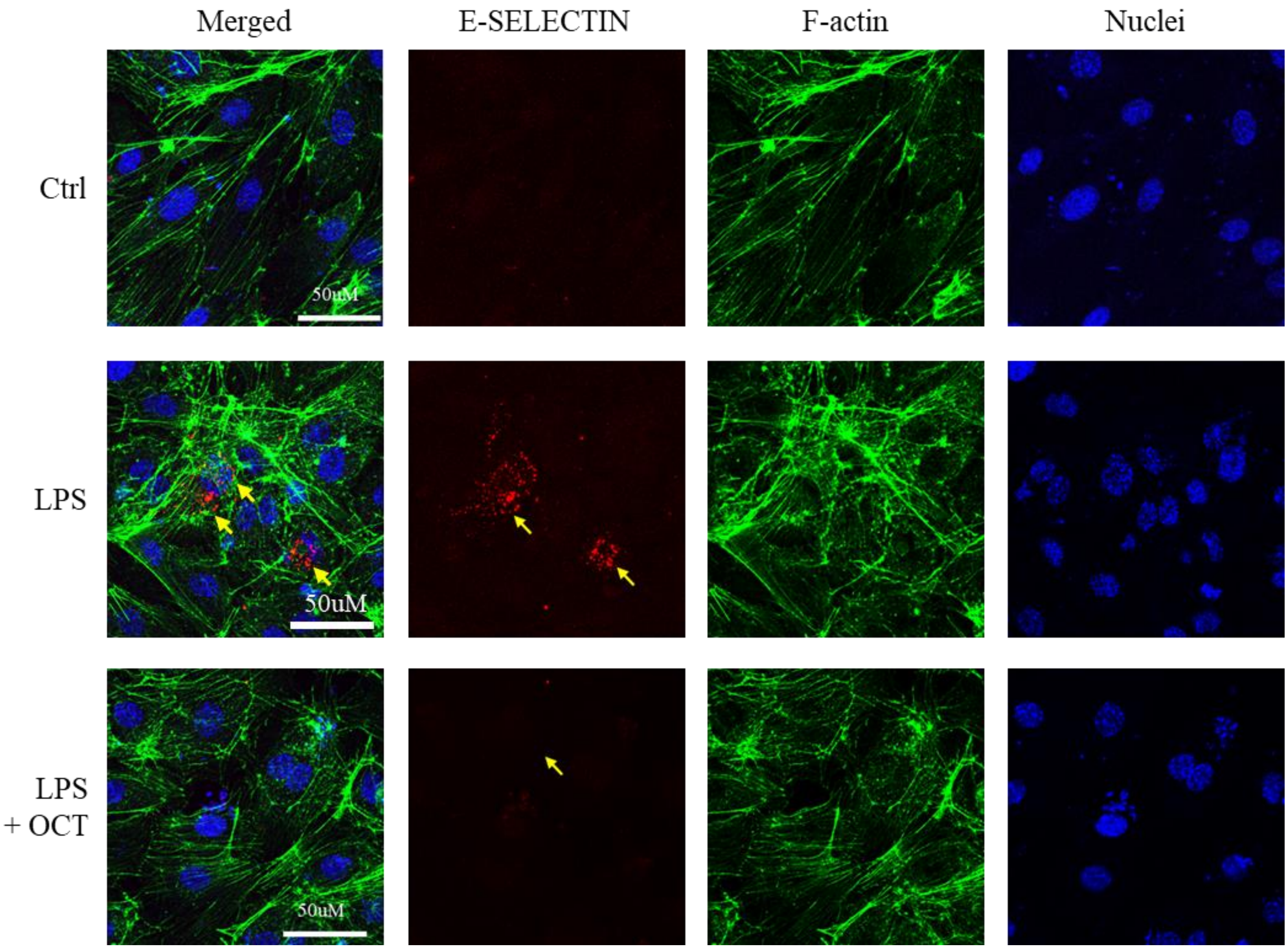

Supplemental Figure 5. Representative confocal image (60X) from HAECs. Panels from left: Merged HAEC image, HAECs labeled against P-SELECTIN (red), F-actin stained HAEC labeling sub-cellular organelles (green), and To-pro-3 stained nuclei (blue). Upper panel (Ctrl): Control HAECs treated with vehicle and without LPS stimulation; middle panel (LPS): HAEC pretreated with vehicle overnight, followed by LPS (100 ng/mL) stimulation for 4 h; bottom panel (LPS+OCT): HAECs pretreated with OCT at 2.5 μM overnight, followed by LPS (100 ng/mL) stimulation for 4 h. Yellow arrows stand for P-SELECTIN clusters under LPS treatment. Ctrl: Control.

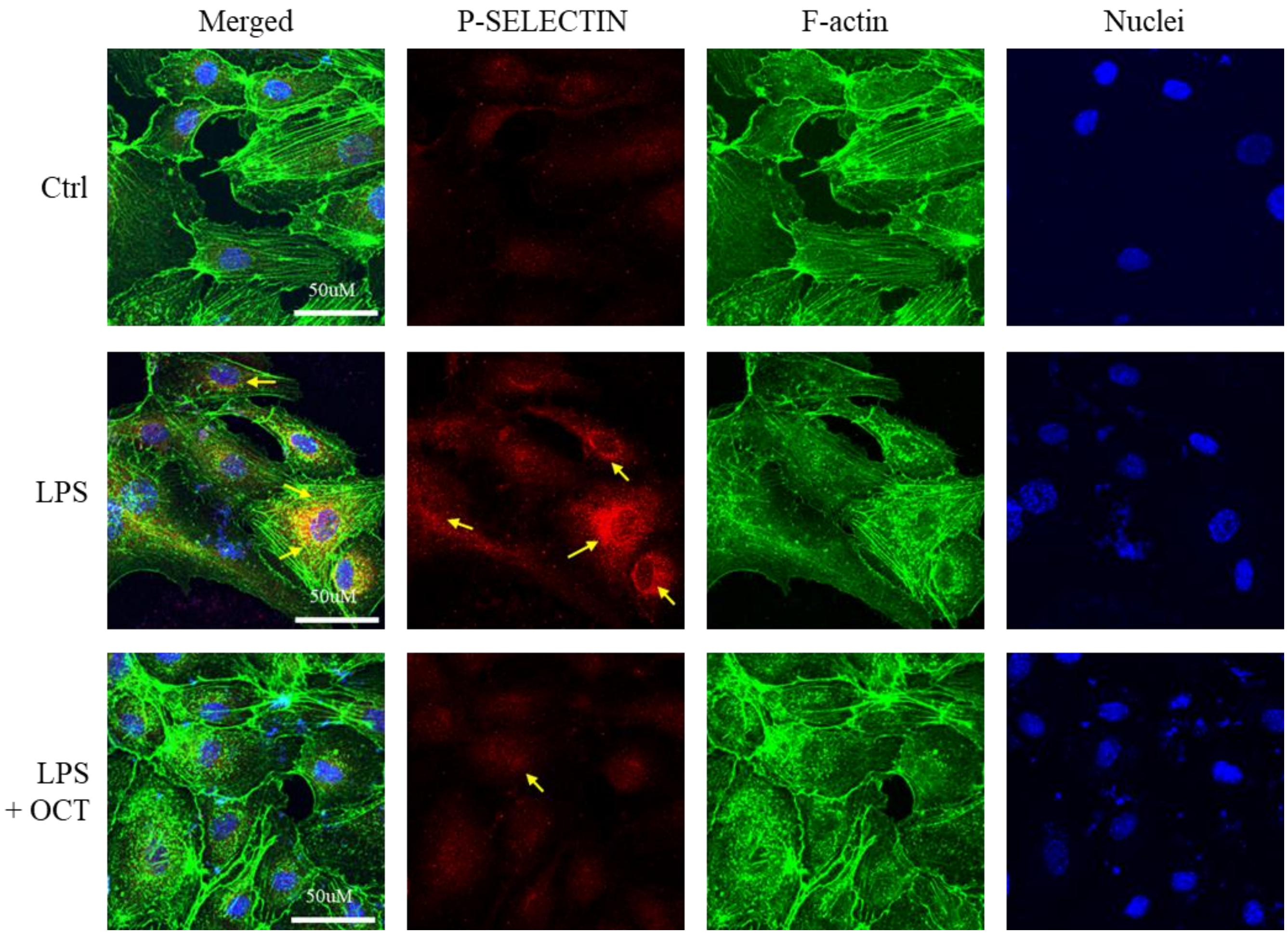

Supplemental Figure 6. Representative confocal image (60X) from HAEC. Panels from left: Merged HAEC image, HAECs labeled against VE-CADHERIN (red), F-actin stained HAEC labeling sub-cellular organelles (green), and To-pro-3 stained nuclei (blue). Upper panel (Ctrl): Control HAECs treated with vehicle and without LPS stimulation; middle panel (LPS): HAECs pretreated with vehicle overnight, followed by LPS (100 ng/mL) stimulation for 4 h; bottom panel (LPS+OCT): HAECs pretreated with OCT at 2.5 µM overnight, followed by LPS (100 ng/mL) stimulation for 4 h. Yellow arrows stand for VE-CADHERIN stained intact around cell border, and white arrows stand for defects of the targeted protein around the cell edges. Ctrl: Control.

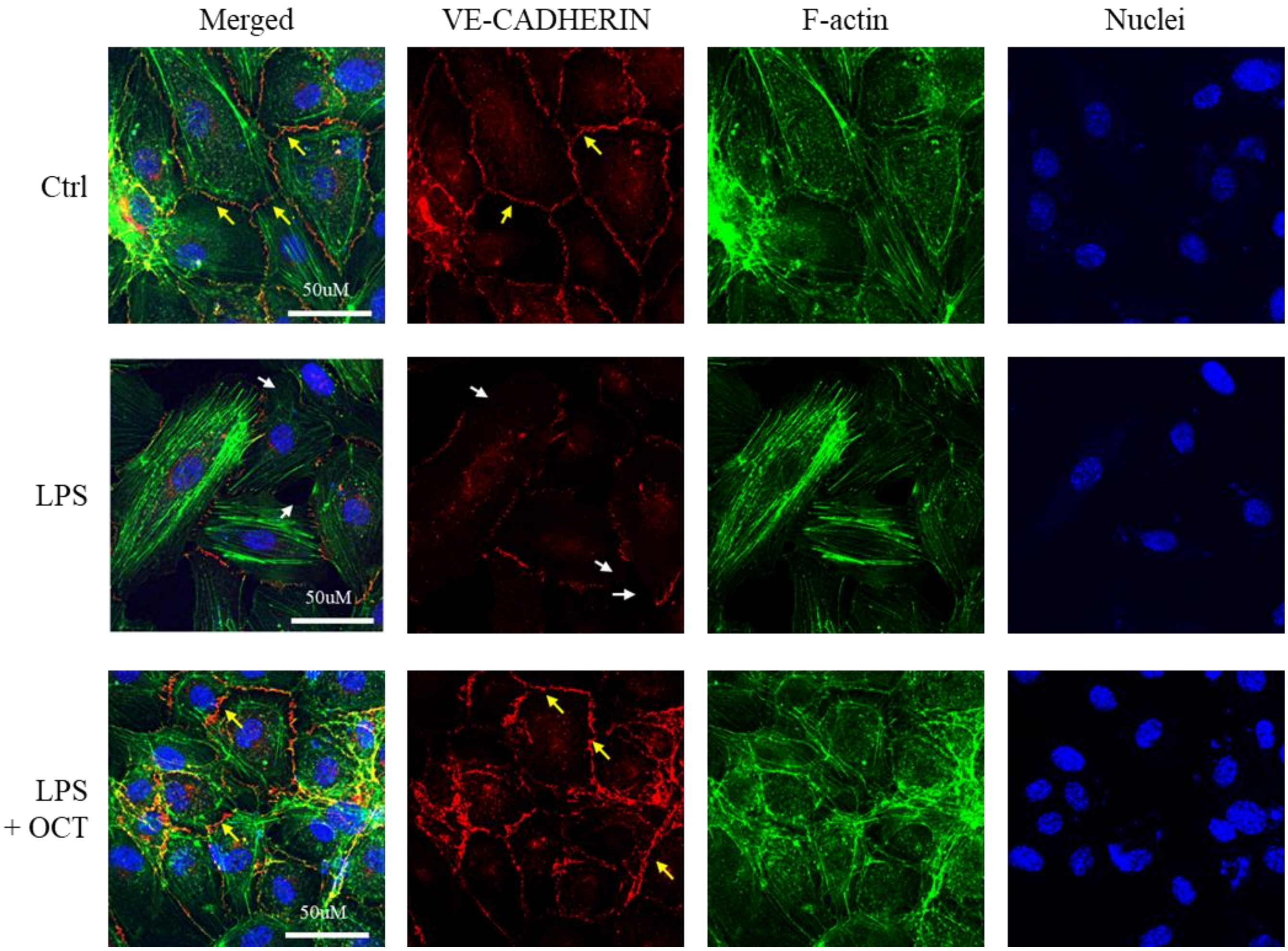

Supplemental Figure 7. Representative confocal image (60X) from HAECs. Panels from left: Merged HAEC image, HAEC labeled against BETA-CATENIN (red), F-actin stained HAEC labeling sub-cellular organelles (green), and To-pro-3 stained nuclei (blue). Upper panel (Ctrl): Control HAECs treated with vehicle and without LPS stimulation; middle panel (LPS): HAEC pretreated with vehicle overnight, followed by LPS (100 ng/mL) stimulation for 4 h; bottom panel (LPS+OCT): HAEC pretreated with OCT at 2.5 µM overnight, followed by LPS (100 ng/mL) stimulation for 4 h. Yellow arrows stand for BETA-CATENIN stained intact around cell border, and white arrows stand for defects of the targeted protein around the cell edge. Ctrl: Control.

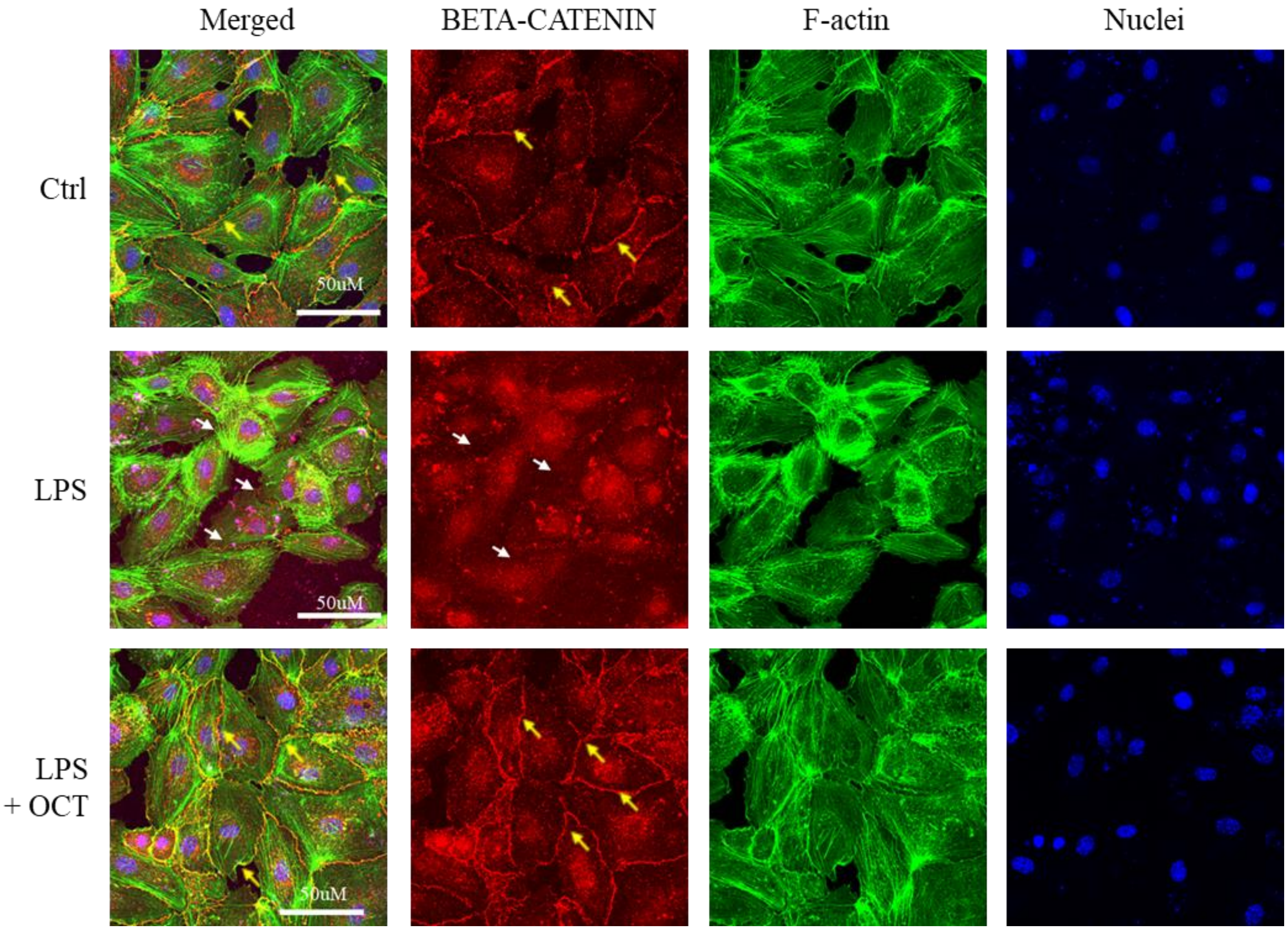

Supplemental Figure 8. Representative confocal image (60X) from HAECs. Panels from left: Merged HAEC image, HAEC labeled against ZO-1 (red), F-actin stained HAEC labeling sub-cellular organelles (green), and To-pro-3 stained nuclei (blue). Upper panel (Ctrl): Control HAECs treated with vehicle and without LPS stimulation; middle panel (LPS): HAEC pretreated with vehicle overnight, followed by LPS (100 ng/mL) stimulation for 4 h; bottom panel (LPS+OCT): HAEC pretreated with OCT at 2.5 μM overnight, followed by LPS (100 ng/mL) stimulation for 4 h. Yellow arrows stand for ZO-1 stained intact around cell border, and white arrows stand for defects of the targeted protein around the cell edges. Ctrl: Control.

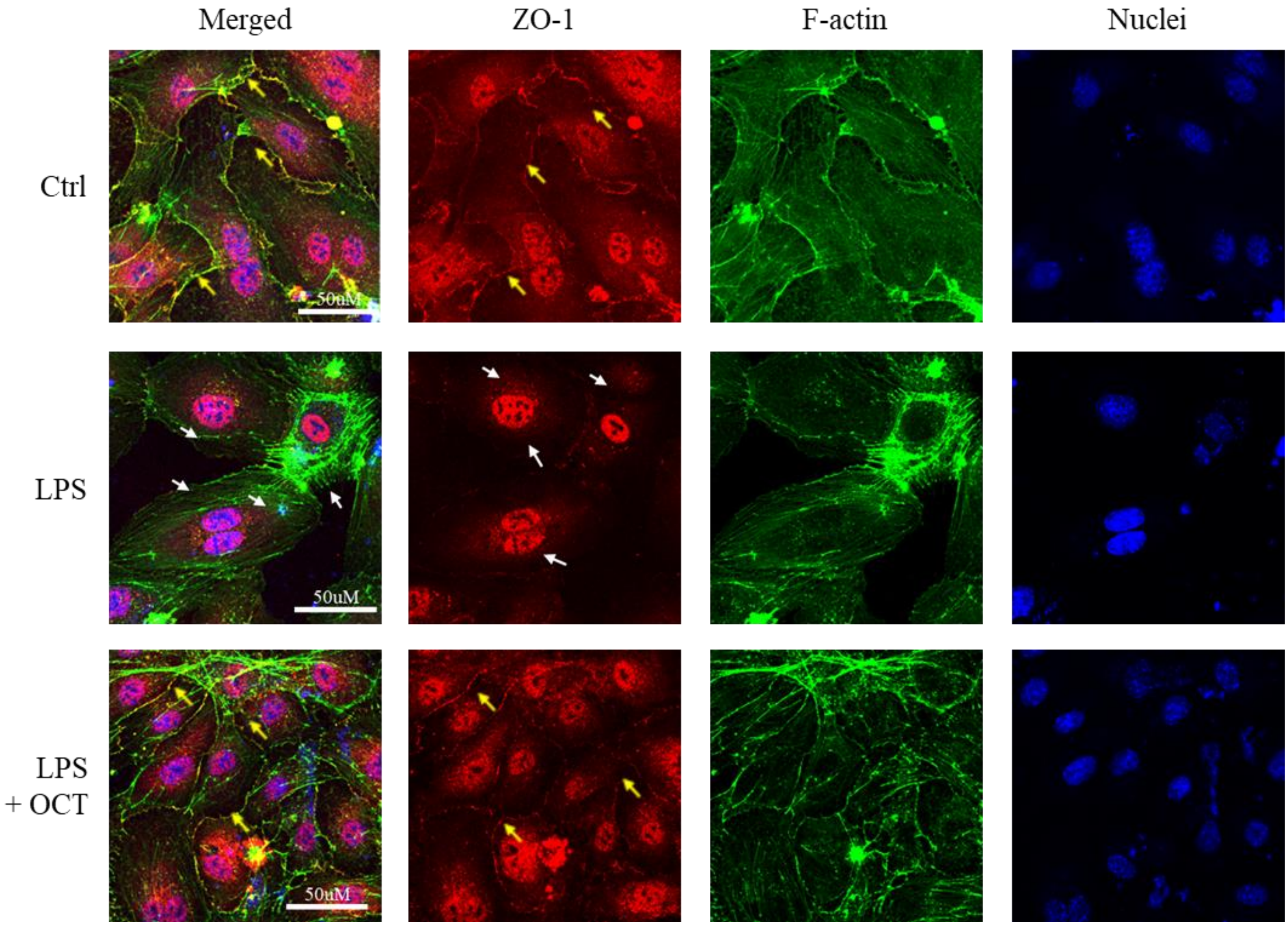

Supplemental Figure 9. Representative confocal image (60X) from HAEC. Panels from left: Merged HAEC image, HAEC labeled against CORTACTIN (red), F-actin stained HAEC labeling sub-cellular organelles (green), and To-pro-3 stained nuclei (blue). Upper panel (Ctrl): Control HAECs treated with vehicle and without LPS stimulation; middle panel (LPS): HAECs pretreated with vehicle overnight, followed by LPS (100 ng/mL) stimulation for 4 h; bottom panel (LPS+OCT): HAEC pretreated with OCT at 2.5 μM overnight, followed by LPS (100 ng/mL) stimulation for 4 h. Yellow arrows stand for CORTACTIN aggregated in cell protrusion under LPS treatment. Ctrl: Control.

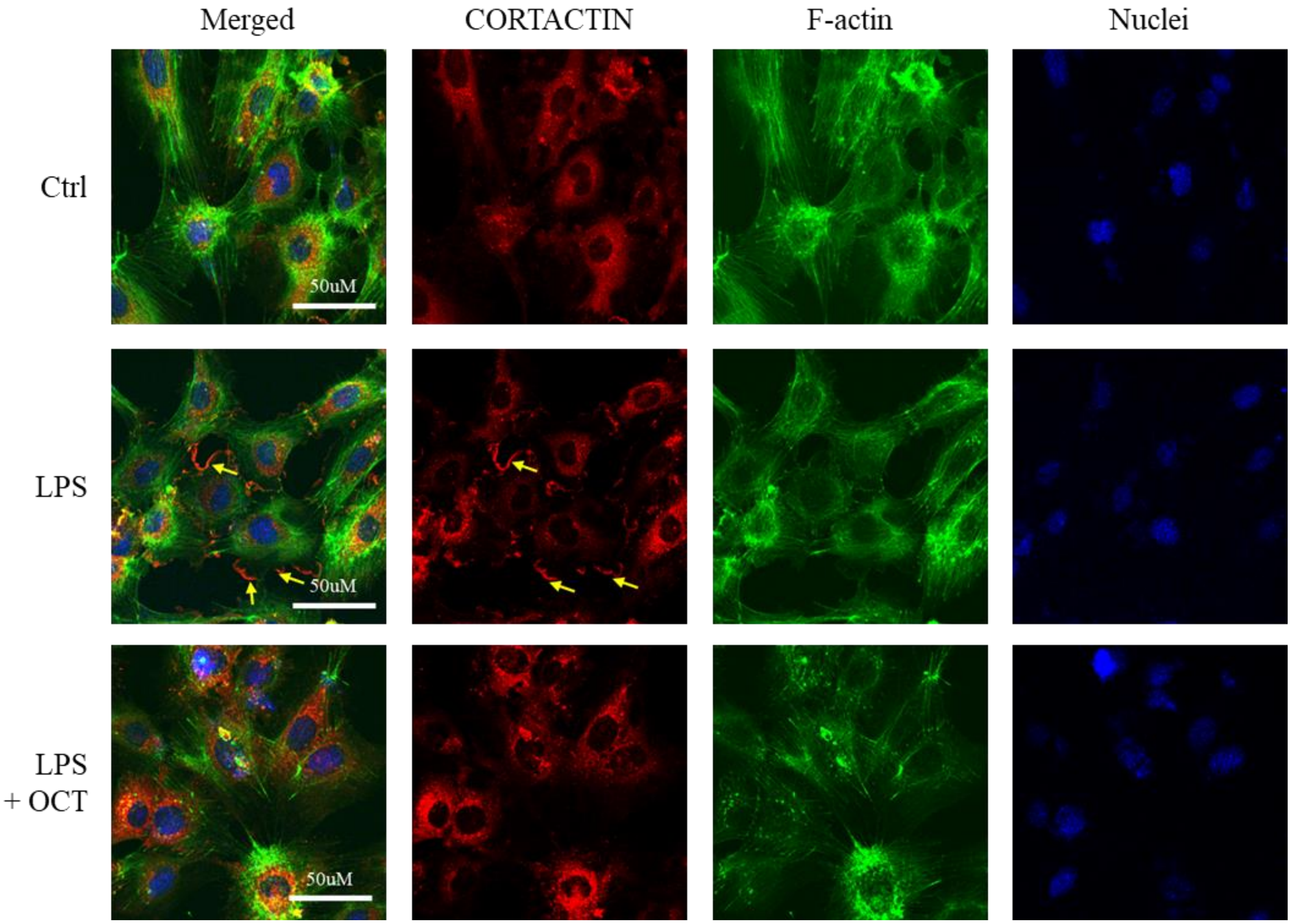

Supplemental Figure 10. Representative confocal image (60X) from HAECs. Panels from left: Merged HAEC image, HAECs labeled against VINCULIN (red), F-actin stained HAEC labeling sub-cellular organelles (green), and To-pro-3 stained nuclei (blue). Upper panel (Ctrl): Control HAECs treated with vehicle and without LPS stimulation; middle panel (LPS): HAECs pretreated with vehicle overnight, followed by LPS (100 ng/mL) stimulation for 4 h; bottom panel (LPS+OCT): HAECs pretreated with OCT at 2.5 μM overnight, followed by LPS (100 ng/mL) stimulation for 4 h. Yellow arrows stand for VINCULIN enriched in cell lamellipodia and show match-head like distribution under LPS treatment. Ctrl: Control.

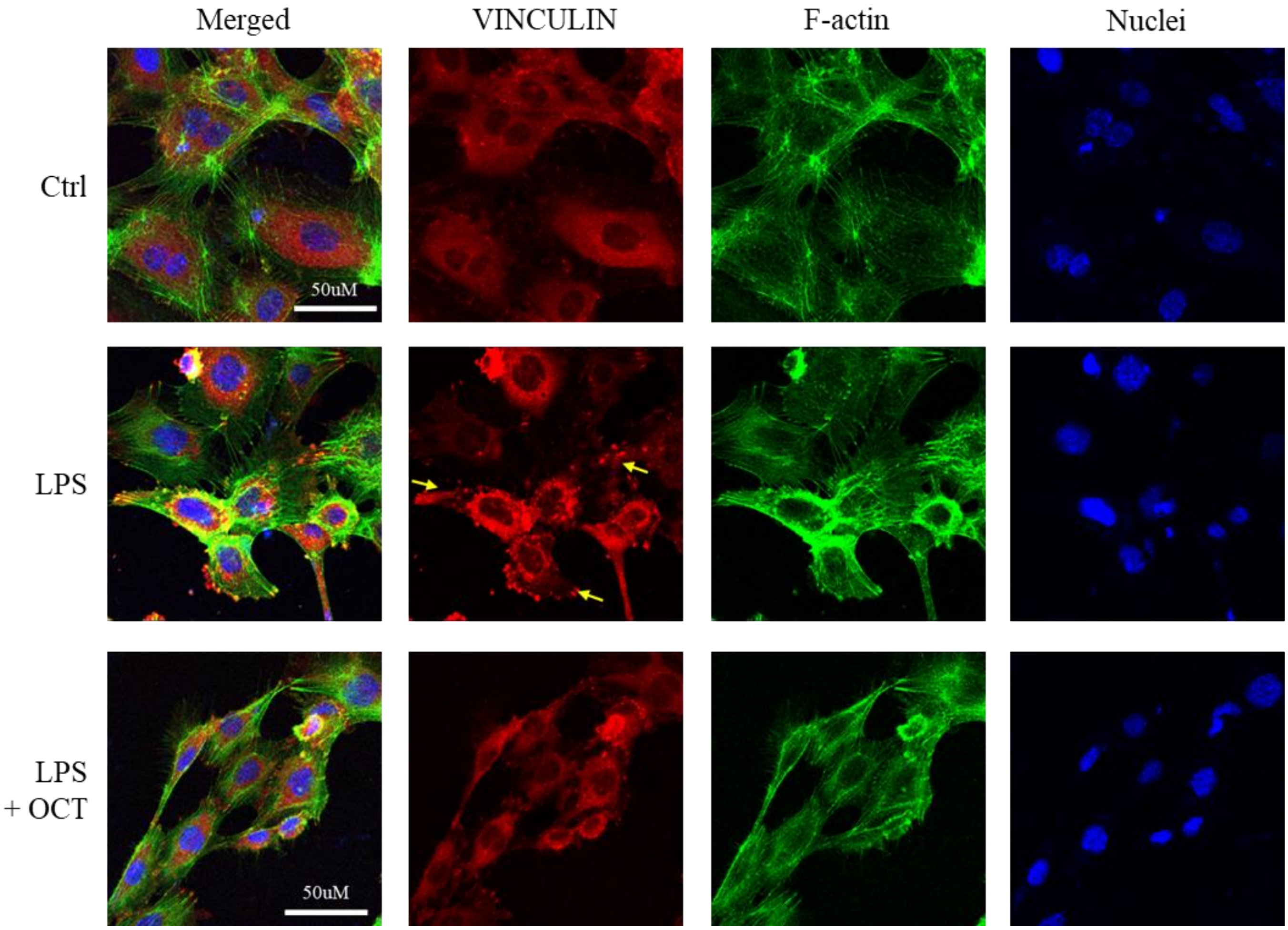

Supplemental Figure 11. Representative confocal image (60X) from HAECs. Panels from left: Merged HAEC image, HAECs labeled against TALIN (red), F-actin stained HAEC labeling sub-cellular organelles (green), and To-pro-3 stained nuclei (blue). Upper panel (Ctrl): Control HAECs treated with vehicle and without LPS stimulation; middle panel (LPS): HAECs pretreated with vehicle overnight, followed by LPS (100 ng/mL) stimulation for 4 h; bottom panel (LPS+OCT): HAECs pretreated with OCT at 2.5 µM overnight, followed by LPS (100 ng/mL) stimulation for 4 h. Yellow arrows stand for TALIN enriched in cell protrusion and show match-head like distribution under LPS treatment. Ctrl: Control.

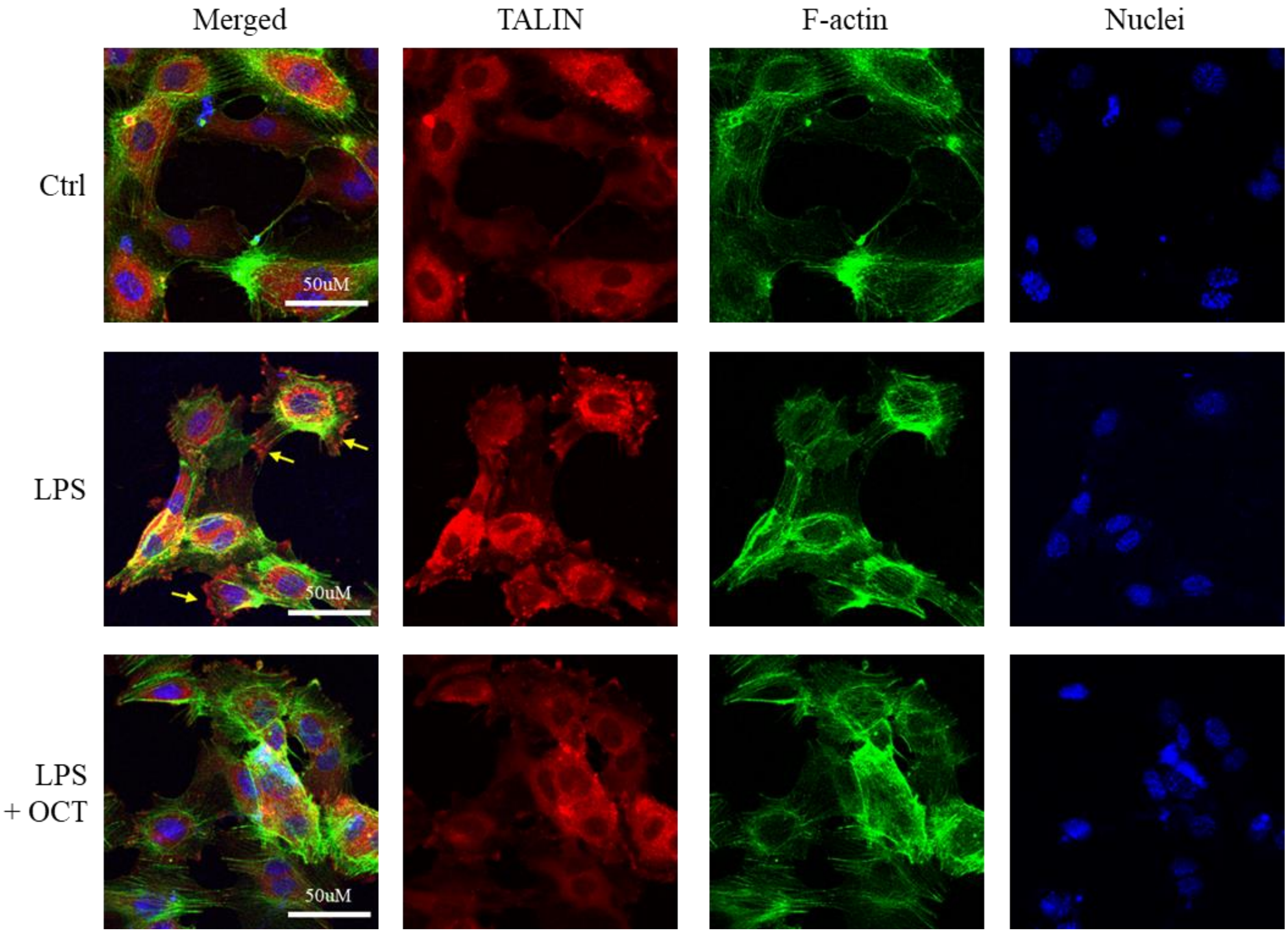

Supplement: Supplementary file 1 [file DataSheet1.pdf]
